# Serotype distribution of invasive and non-invasive pneumococcal disease in adults ≥65 years of age following the introduction of 10- and 13-valent pneumococcal conjugate vaccines in infant national immunization programs: a systematic literature review

**DOI:** 10.3389/fpubh.2025.1544331

**Published:** 2025-05-30

**Authors:** Patricia Izurieta, Dorota Borys

**Affiliations:** Vaccines R&D/Infectious Disease, GSK, Wavre, Belgium

**Keywords:** invasive pneumococcal disease, non-invasive community-acquired pneumonia, older adults, pneumococcal conjugate vaccine, serotype distribution, *Streptococcus pneumoniae*

## Abstract

**Introduction:**

Despite the widespread implementation of 10- and 13-valent pneumococcal conjugate vaccines (PCVs) in infant national immunization programs and anticipated herd effects, pneumococcal disease incidence remains relatively high among older adults. In this vulnerable population, this includes not only invasive pneumococcal disease (IPD), but, more notably, non-invasive community-acquired pneumonia (CAP). A comprehensive understanding of adult pneumococcal epidemiology, particularly that of non-invasive CAP, is essential to guide future vaccination strategies for this population.

**Methods:**

We systematically reviewed observational studies (2006–2020) on pneumococcal serotype distribution in IPD and non-invasive CAP among adults aged ≥65 years after PCV implementation in children, focusing on the period post-implementation of the 10-valent pneumococcal non-typeable *Haemophilus influenzae* protein D conjugate vaccine (PHiD-CV) and 13-valent PCV (PCV13). Serotype-specific pooled percentage averages were calculated to determine the contribution of each serotype to a certain clinical manifestation.

**Results:**

Our analysis of 17 IPD and 17 CAP studies indicates the persistence of several vaccine serotypes, particularly serotypes 3 and 19A, in both clinical manifestations. Also serotype 7F remained frequently reported. The predominant non-PCV13 serotypes identified in both manifestations were serotypes 8, 12F, 15A, and 22F.

**Conclusion:**

The persistence of certain PCV13-serotypes in pneumococcal disease among adults aged ≥65 years suggests that herd immunity by infant PCV immunization may be insufficient to provide optimal protection in this population. This, coupled with emerging non-PCV13 serotypes due to serotype replacement and other limitations of current vaccines, supports the need for new vaccination technologies and strategies to improve protection of older adults.

## 1 Introduction

*Streptococcus pneumoniae* (Spn) causes significant morbidity and mortality globally, particularly among young children (aged ≤5 years), older adults (aged ≥65 years), and those with underlying medical conditions (1–4). The clinical spectrum of pneumococcal infections ranges from mild conditions such as acute otitis media (AOM) (among children) or sinusitis, to severe conditions like non-invasive community-acquired pneumonia (CAP) and invasive pneumococcal disease (IPD) (3, 5). At least 100 Spn serotypes are known (6), but only a subset of these is responsible for most infections (7).

Pneumococcal conjugate vaccines (PCVs) targeting particular serotypes have been widely used in infant national immunization programs (NIPs) for nearly 2 decades. Starting from 2009, the 7-valent PCV (PCV7, *Prevenar/Prevnar*, Pfizer Inc.) was replaced in infant NIPs by the 10-valent pneumococcal non-typeable *Haemophilus influenzae* protein D conjugate vaccine (PHiD-CV, *Synflorix*, GSK) or the 13-valent PCV (PCV13, *Prevenar 13/Prevnar 13*, Pfizer Inc.) (7). Since 2015, PCV uptake in infants has been high in high-income countries, with uptake levels exceeding 80%, while uptake has generally been lower in low- and middle-income countries, ranging between 28 and 60% (8). With the recent authorization of new PCVs for infant use, targeting 15 (PCV15, *Vaxneuvance*, Merck Sharp & Dohme LLC, a subsidiary of Merck & Co., Inc. [MSD]) or 20 (PCV20, *Prevenar 20/Prevnar 20*, Pfizer Inc.) serotypes, health authorities are currently re-evaluating their recommendations (9).

For adults, guidelines on pneumococcal vaccination vary among countries. Since the 1980s, the pneumococcal polysaccharide vaccine covering 23 serotypes (PPSV23, *Pneumovax 23*, Merck Sharp & Dohme LLC, a subsidiary of Merck & Co., Inc. [MSD]) has been widely recommended for high-risk individuals aged ≥2 years (10), and many countries also recommend the vaccine for adults aged ≥65 years (11–14). However, PPSV23 has been shown to elicit an immune response that is neither long lasting nor anamnestic upon subsequent challenge (15), and its effectiveness has been estimated at 45–59% against IPD, and 48–53% against pneumococcal pneumonia by cohort and case–control studies (16). Since 2014, some countries recommend PCV13 for use in vulnerable adult populations (9) and are currently considering PCV15, PCV20 or the recently approved 21-valent PCV (PCV21, *Capvaxive*, Merck Sharp & Dohme LLC, a subsidiary of Merck & Co., Inc. [MSD]) (9, 17).

Although PCV use has significantly reduced pneumococcal disease incidence in children (18–20), and despite the potential benefits of both direct adult vaccination and herd protection in adults from infant PCV programs, the burden of pneumococcal diseases remains high among older adults (21–26). One reason may be that the pneumococcal vaccine uptake among older adults has remained suboptimal, even in high-income countries, with average rates of 68% in the United States (US) (2019–2020), 58% in Canada (2018–2019), 33.6% in Japan (2014–2020), and just 18% in Europe (2015–2016) (27–30). Besides, the remaining burden may be explained by inadequate (herd) protection of current vaccines against certain serotypes, and differences in serotype distribution between age groups. Moreover, serotype replacement, a phenomenon where the reduction in PCV-targeted serotypes leads to increased circulation and disease caused by non-PCV serotypes (23, 31), may amplify the burden. Therefore, understanding adult pneumococcal disease epidemiology after PHiD-CV/PCV13 implementation in infant NIPs is essential to guide future vaccination strategies.

Pneumococcal epidemiological data are primarily available on IPD because serotyping is a routine component of most mandatory IPD surveillance programs (7). However, the serotype distribution may vary by clinical manifestation of Spn (7, 32–35). Understanding the epidemiology of non-invasive pneumococcal CAP is thus at least equally important, given its higher incidence among older adults compared to IPD and the substantial associated healthcare costs (24, 36). Still, serotype data for this clinical manifestation remain relatively scarce (7), largely due to limitations in available microbiological methods, such as the low sensitivity of sputum cultures and sputum PCR due to poor sample quality and prior antibiotic use (37), and the incomplete serotype coverage of urinary antigen detection tests and immunoassays (38–40). Additionally, no dedicated surveillance mechanisms exist for non-invasive CAP (7, 24).

In this systematic literature review (SLR), our objective was to synthesize worldwide published data of observational studies on pneumococcal serotype distribution in both IPD and non-invasive CAP in adults aged ≥65 years after the widespread uptake of PHiD-CV/PCV13 in infants, compared to the post-PCV7 era. Ultimately, this review aimed to enhance our understanding of the dynamic interplay between PHiD-CV/PCV13 implementation in infant NIPs, herd protection, serotype replacement, and the pneumococcal epidemiology in older adults.

## 2 Methods

The present analysis is part of a larger SLR that aimed to evaluate the impact of infant PHiD-CV/PCV13 uptake on the serotype distribution in remaining invasive and non-invasive pneumococcal disease in both children aged ≤5 years and adults aged ≥65 years. The pneumococcal diseases of interest were AOM (among children only), CAP, and IPD. This manuscript summarizes the findings for adults. The findings for children are discussed in a separate manuscript (41). The review was performed according to a predefined protocol and followed the Preferred Reporting Items for Systematic Reviews and Meta-Analyses (PRISMA) guidelines (42).

### 2.1 Systematic search strategy

PubMed and Embase were systematically searched to retrieve articles on pneumococcal serotype distribution in IPD and CAP among adults aged ≥65 years after the implementation of PCV7, PHiD-CV, or PCV13 in infant NIPs. Search strings consisted of a combination of terms for Spn serotypes, PCVs, and pneumococcal diseases. A limitation was set on the date of publication from 1 January 2006 to 31 December 2020, considering the widespread utilization of PCV7 in infant NIPs in many Western countries in 2006, while also limiting the extraction of data that was obtained during and after the coronavirus disease 2019 (COVID-19) pandemic (COVID-19 was declared a pandemic by the WHO in March 2020). Post-PHiD-CV/PCV13 serotyping data were of primary interest to our study. Studies conducted after PCV7 implementation were included to assess changes in serotype distribution before and after PHiD-CV/PCV13 uptake. Additional details can be found in the Supplementary methods.

### 2.2 Eligibility criteria and study selection

All predefined eligibility criteria for study inclusion were applied at 2 stages: at screening and at the data extraction phase. For this manuscript, studies including data on adults ≥65 years were retained.

Eligible studies related to CAP were observational studies. CAP was defined as pneumonia acquired outside of the hospital (43), and serotyping was done on samples obtained from non-sterile sites (sputum, urine, bronchial aspirates, biopsy samples, etc.). Studies on invasive CAP were not eligible given that this condition is typically classified under IPD. To minimize the potential impact of incidental small findings or limited cohort observations, only CAP studies reporting on a predefined minimum of 20 serotyped isolates were included.

Given that many published observational studies on IPD were expected, eligible IPD studies were in the first instance limited to SLRs/meta-analyses. Since no SLRs were identified, as predefined in the protocol, the eligibility criteria for IPD were expanded to include the most recent pre-COVID-19 observational studies published between 2018 and 2020. Serotyping was done on samples obtained from sterile sites (blood, cerebrospinal fluid, pleural effusion, joint fluid, pericardial fluid, etc.) (44). Only studies reporting on at least 30 serotyped isolates were included, as many publications were expected for IPD allowing for more stringent criteria compared to CAP.

A detailed description of inclusion/exclusion criteria and study selection workflow is outlined in the Supplementary methods.

### 2.3 Data extraction and analysis

To determine the contribution of each serotype to a certain clinical manifestation, pooled percentage averages were calculated for each serotype using the following formula:


$$\frac{\text{sum}\;(\text{number of samples}\;\text{per}\;\text{serotype})}{\text{sum}\;(\text{total number of samples serotyped})\;}\times 100$$

where the “sum” corresponds to the total number of samples across studies included in the corresponding analysis. The serotype-specific pooled percentage average thus determines the average proportion of a given serotype, relative to all serotyped samples of the studies included in the corresponding analysis. This method ensures the weighted representation of the distribution of serotypes in pneumococcal disease (either IPD or CAP) by accounting for variations in sample sizes across the included studies. Since percentages are calculated for each serotype separately, rather than as proportions of a whole, the sum of all pooled percentage averages may exceed 100% for a certain clinical manifestation.

For each clinical manifestation, the included studies were categorized into 2 vaccine periods based on information provided in the publications: post-PCV7 or post-PHiD-CV/PCV13 (pooled), referring to the use of these vaccines in infants.

The primary analysis for each clinical manifestation included only data originating from studies conducted in countries where the PCV was implemented through infant NIPs. Studies conducted in countries where the PCV was only available in private markets, and not included in the infant NIP, were excluded. Such exclusions were based on the premise that these settings might lack the effects of herd protection due to low infant PCV uptake. Additionally, a more comprehensive sensitivity analysis was conducted for each clinical manifestation, based on data from all eligible studies, including those in countries that implemented PCVs in the private market only. Furthermore, subgroup analyses were conducted by classifying each eligible study based on the PCV product (PHiD-CV or PCV13).

Serotype-specific pooled percentage averages were reported only if data from at least 5 studies were available for that serotype. This threshold was established to limit the potential for data skewing resulting from a limited number of studies, focusing on the serotypes supported by stronger evidence. This criterion could not be applied to all subgroup analyses per PCV product and the post-PCV7 analyses due to the limited number of eligible studies for some subgroups.

The analyses were performed using R studio. All analyses were descriptive, and no formal statistical testing was performed. More details on the data extraction procedure and study categorization can be found in the Supplementary methods.

## 3 Results

### 3.1 Study selection

**Figure 1:**
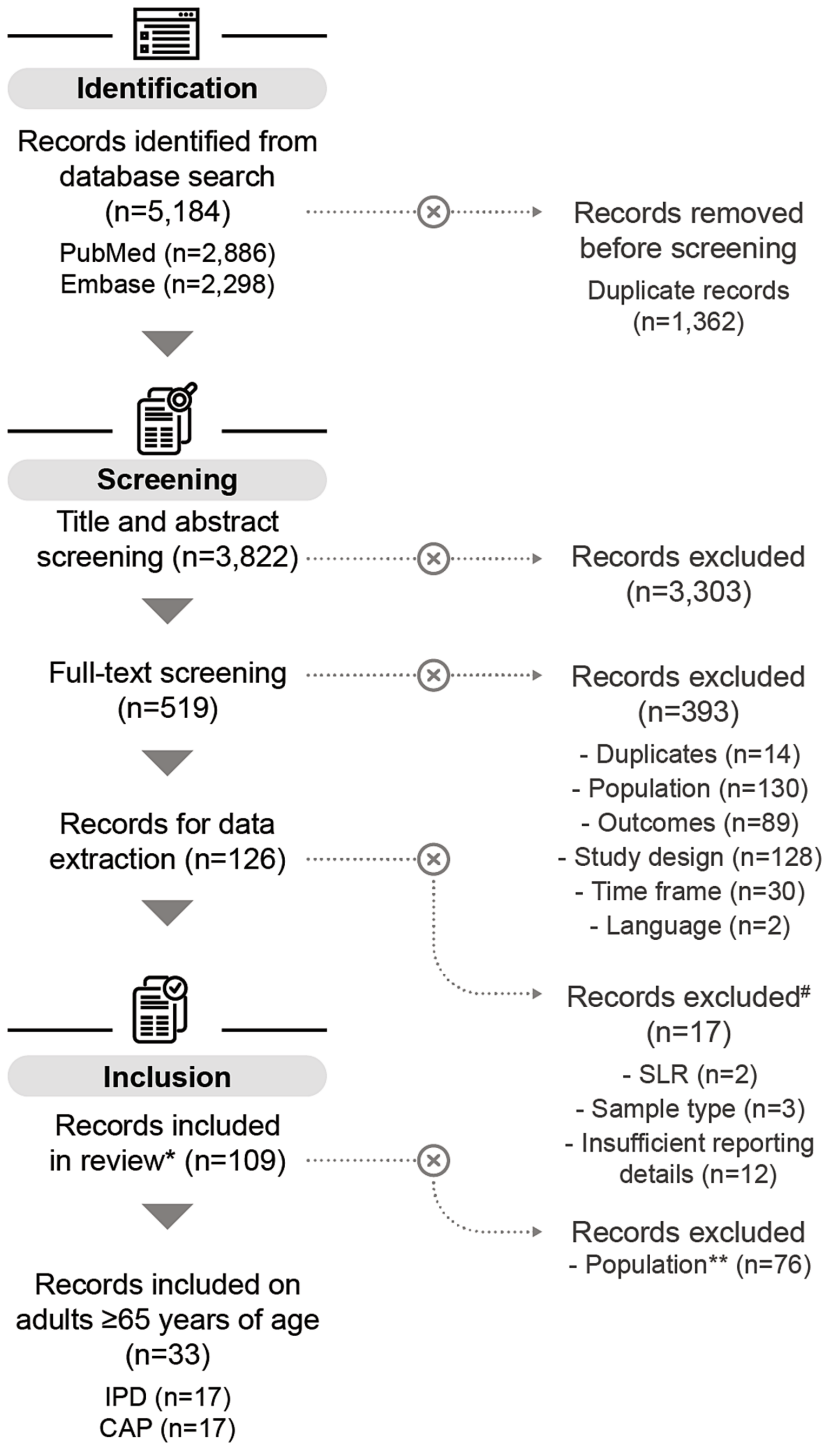
PRISMA flow chart of the systematic literature search. # All records excluded during data extraction relate to children aged ≤5 years. *Some of the records included in this review also contain data on children aged ≤5 years (*n* = 11), but these datapoints were not included in the analysis on adults aged ≥65 years. One study on adults aged ≥65 years reported on both IPD and CAP. **Records included in this review containing only data on children aged ≤5 years were excluded for the purpose of this publication. n, number of records; SLR, systematic literature review; IPD, invasive pneumococcal disease; CAP, community-acquired pneumonia.

Of the 3,822 screened publications, 126 studies were selected for data extraction, of which 109 studies were included in the quantitative analysis. The main reason for study exclusion was the lack of sufficient details for further analysis. In total, 33 studies covered serotype distribution data in adults aged ≥65 years. Of these, 17 reported data on IPD (45–61), and 17 on non-invasive CAP (49, 62–77); [one study reported on both disease manifestations (49)] (Figure 1). Of the 33 studies considered, most were conducted in Europe (*n* = 18), followed by Asia (*n* = 10), North America (*n* = 4), and South America (*n* = 1).

### 3.2 IPD

#### 3.2.1 IPD study characteristics

Across all included studies on IPD, 4/17 studies reported data obtained post-PCV7 infant implementation (51, 54, 57, 58), and 14/17 post-PHiD-CV/PCV13 uptake (45–50, 53–56, 58–61). There were 2 studies that reported stratified data following both PCV7 and PHiD-CV or PCV13 (54, 58). One study reported data obtained post-PCV7 and post-PCV13 implementation without differentiating which period the data corresponded to, and could thus not be used for further analysis (52). The majority of the post-PHiD-CV/PCV13 studies were conducted in infant NIP settings (12/14 studies), and most exclusively used PCV13 (12/14 studies). Most of these PCV13 studies (8/12 studies) included data that were collected up to 4 to 8 years following its introduction in infant NIPs. One study was in a setting of exclusive PHiD-CV use [Austria (59)], and 1 in a setting of mixed PHiD-CV and PCV13 use [South Korea (53)]. In total, serotyping data from 27,757 adult IPD isolates were extracted post-PHiD-CV/PCV13 uptake in infants. Study-specific details, including study type, age range, sample type, total number of isolates, PCV product, inclusion in the infant NIP, and the range of years of PCV use, are provided in Table 1.

**Table 1:** Characteristics of the included IPD studies post-PHiD-CV/PCV13 uptake in infants.

Author, year (ref)	Study design	Country	Median age	Age range	Sample type	N	Data collection period	PHiD-CV/ PCV13 for infants	Infant NIP*	Years of PHiD-CV/PCV13 use via infant NIP
IPD										
*n* = 14						27,757			*n* = 12	
Amin-Chowdhury et al. (45)	Prospective cohort	UK	66Y	49–67Y	Blood/CSF/Pleural effusion/Sterile site	9,098	2014/2015–2017/2018	PCV13	Yes	8
Ciruela et al. (46)	Retrospective cohort	Spain	NR	65–74Y 75-84Y ≥85Y	Blood/CSF/Joint fluid/Pericardial fluid/Peritoneal fluid/Lung tissue	1,114	2009; 2014–2016	PCV13	Yes	2
Danis et al. (47)	Surveillance	France	71Y	65–84Y ≥85Y	Blood/Joint fluid/Pleural effusion/Peritoneal fluid	587	2014–2017	PCV13	Yes	7
de Miguel et al. (48)	Surveillance	Spain	NR	≥65Y	NR	1,239	2009–2019	PCV13	Yes	3
Kim et al. (49)	Surveillance	South Korea, China, Malaysia, Philippines, Singapore, Thailand	NR	≥50Y	Blood/CSF/Pleural effusion/Sterile site	245	December 2012 –July 2017	PCV13	No	NA
Ladhani et al. (50)	Prospective cohort	UK	NR	≥65Y	Sterile site	2,947	2000–2001; 2016–2017	PCV13	Yes	7
Park et al. (53)^a^	Cross-sectional	South Korea	NR	≥65Y	Blood/CSF/Pleural effusion	205	May 2014–May 2016	PHiD-CV/ PCV13	Yes	2
Ubukata et al. (54)	Surveillance	Japan	NR	≥65Y	Sterile site	880	April 2010–March 2017	PCV13	Yes	4
Yanagihara et al. (55)	Prospective cohort	Japan	70Y	23–101Y	Blood/CSF/Pericardial fluid/Pleural effusion/Sterile site/Bone	177	September 2016–December 2018	PCV13	Yes	5
Zintgraff et al. (56)	Surveillance	Argentina	NR	≥65Y	Blood/CSF/Joint fluid/Pleural effusion/Sterile site/Bone/ Placenta/Skin/Ascitic fluid	791	2013–2017	PCV13	Yes	5
Ciruela et al. (58)	Retrospective cohort	Spain	NR	≥65Y	Blood/CSF/Pleural effusion/Sterile site	2,043	2006–2014	PCV13	No	NA
Richter et al. (59)	Before-after study	Austria	NR	≥65Y	Sterile site	608	2009–2017	PHiD-CV	Yes	5
Fenoll et al. (60)	Prospective cohort	Spain	NR	≥65Y	Blood/CSF/Pleural effusion/Sterile site/Ascitic fluid	542	August 2010–June 2015	PCV13	Yes	0
Demczuk et al. (61)	Surveillance	Canada	NR	65–109Y	Blood/CSF/Joint fluid/ Pericardial fluid/Pleural effusion/Sterile site/Peritoneal fluid/Deep abscesses, and tissues fluid	7,281	2010–2016	PCV13	Yes	5

a Excluded from stratified analysis per PCV product because PHiD-CV and PCV13 were used simultaneously at the time of the study.

* Studies included in the primary post-PHiD-CV/-PCV13 analysis. CSF, cerebrospinal fluid; IPD, invasive pneumococcal disease; N, total number of serotyped isolates; n, number of studies; NA, not applicable; NIP, national immunization program; NR, not reported; PCV, pneumococcal conjugate vaccine; PCV13, 13-valent PCV; PHiD-CV, pneumococcal non-typeable *Haemophilus influenzae* protein D conjugate vaccine; ref, reference; UK, United Kingdom; Y, years.

#### 3.2.2 Serotype distribution in adult IPD post-PHiD-CV/PCV13 uptake in infants

**Figure 2:**
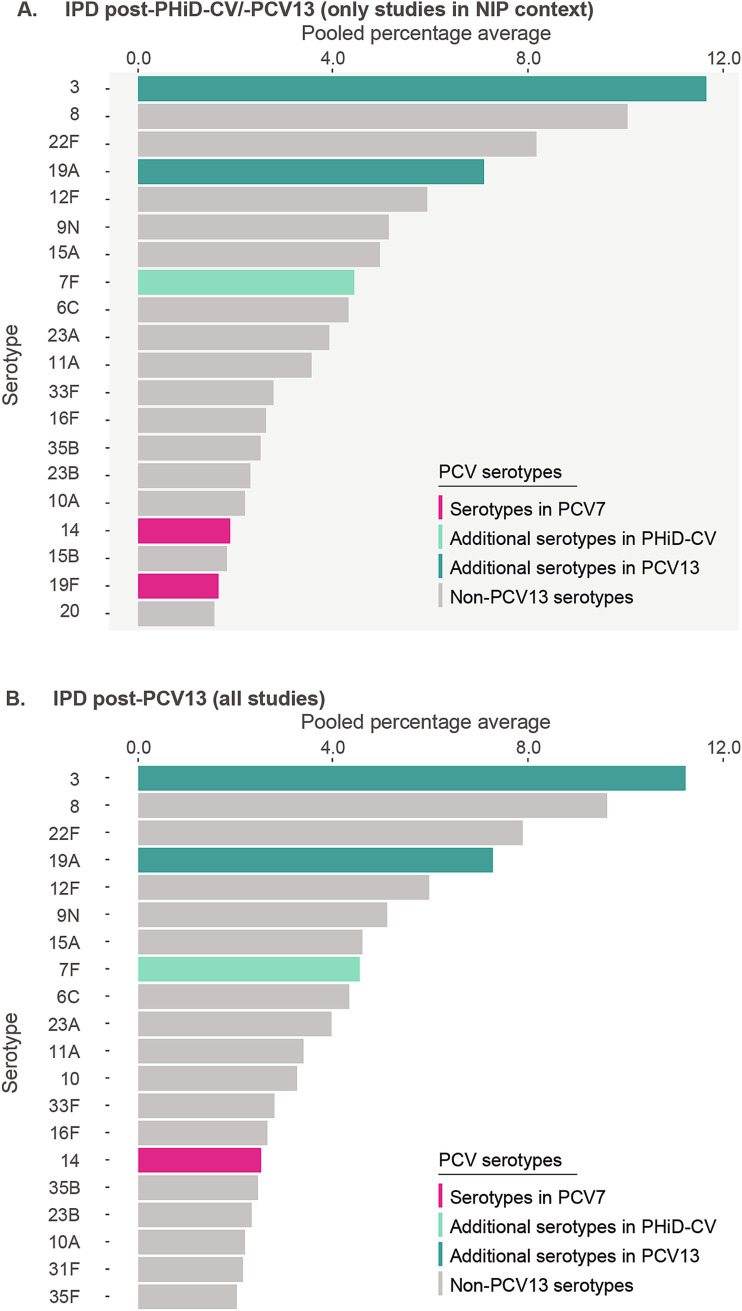
Serotype distribution in invasive pneumococcal disease among adults aged ≥65 years **(A)** post-PHiD-CV/PCV13 implementation through infant national immunization programs (*n* = 12), and **(B)** post-PCV13 uptake in infants (either through infant national immunization programs or private markets) (*n* = 12). The top 20 serotypes are shown. Serotypes are represented by colors corresponding to the lowest valency PCV product in which they are included. In the PCV legend, the additional serotypes included in the product are relative to the next lower valency product. Pooled percentage averages were calculated for each serotype individually, thus the sum of all serotypes may exceed 100%. For panel A, serotype-specific pooled percentage averages were calculated only if 5 or more studies reported on the respective serotype. For panel B, the pooled percentage averages were calculated irrespective of the number of studies reporting on it. IPD, invasive pneumococcal disease; n, number of studies that were included in the analysis; NIP, national immunization program; PCV, pneumococcal conjugate vaccine; PCV7, 7-valent PCV; PCV13, 13-valent PCV; PHiD-CV, pneumococcal non-typeable *Haemophilus influenzae* protein D conjugate vaccine.

Post-PHiD-CV/PCV13 implementation through infant NIPs (primary analysis comprising data of 12 studies), the 10 most reported serotypes in older adults with IPD were serotype 3, with a pooled percentage average of 11.6% across studies, 8 (10.0%), 22F (8.2%), 19A (7.1%), 12F (5.9%), 9 N (5.1%), 15A (5.0%), 7F (4.4%), 6C (4.3%), and 23A (3.9%) (Figure 2A; Supplementary Table S3). A nearly identical serotype distribution pattern was obtained by the sensitivity analysis including 2 additional IPD-related studies where PCV13 was introduced via the private market only (Supplementary Figure S1).

A stratified analysis on data obtained from the 12 PCV13-related studies yielded a highly comparable serotype distribution pattern and corresponding pooled percentage averages (Figure 2B). A single study in PHiD-CV setting (59) reported serotype 3 (28.6%) as the leading serotype, followed by serotypes 19A (7.4%), and 22F (6.1%) (Supplementary Figure S2).

In the post-PCV7 period (3 studies in infant NIP context), serotype 3 (16.6%) was the leading serotype (Supplementary Figure S3). When comparing the reporting of PCV13 serotypes post-PHiD-CV/PCV13 uptake with the post-PCV7 period, the pooled percentage averages in the primary analysis for serotype 3 were 16.6% post-PCV7 vs. 11.6% post-PHiD-CV/PCV13, and 8.6% vs. 7.1% for serotype 19A. An important decrease in the reporting of several other PCV13 serotypes was observed.

### 3.3 Non-invasive CAP

#### 3.3.1 CAP study characteristics

Among the 17 included CAP studies, 3 reported discernible data obtained post-PCV7 infant uptake (67, 72, 76), and 11 post-PHiD-CV/PCV13 uptake (49, 62–64, 66, 68–71, 73, 76). There was one study that reported stratified data from both PCV periods (76). Four studies reported indistinguishable data from both PCV periods and could thus not be used for further analysis (65, 74, 75, 77). All of the studies with data from the post-PHiD-CV/-PCV13 period (11 studies) were in a setting of PCV13 use, and most of these (8/11 studies) were conducted in countries with PCV13 implementation through infant NIPs. Six of these 8 PCV13 studies included data that were collected up to 4 to 8 years following its introduction in infant NIPs. Overall, serotyping data from a total of 3,247 adult CAP isolates were extracted post-PCV13 uptake in infants. Study-specific details, including study type, age range, sample type, total number of isolates, PCV product, inclusion in the infant NIP, and the range of years of PCV use, are provided in Table 2.

**Table 2:** Characteristics of the included non-invasive CAP studies post-PHiD-CV/PCV13 uptake in infants.

Author, year (ref)	Study design	Country	Median age	Age range	Sample type	N	Data collection period	PHiD-CV/ PCV13 for infants	Infant NIP*	Years of PHiD-CV/PCV13 use via infant NIP
CAP										
*n* = 11						3,247			*n* = 8	
Forstner et al. (62)	Prospective cohort	Germany	69Y	51–74Y	Sputum/Urine	59	December 12, 2012–January 26, 2017	PCV13	Yes	8
Kim et al. (49)	Surveillance	South Korea, China, Malaysia, Philippines, Singapore, Thailand	NR	≥50Y	Sputum	448	December 2012–July 2017	PCV13	No	3
LeBlanc et al. (63)	Surveillance	Canada	76Y	65–103Y	Sputum/Urine	257	2010–2015	PCV13	Yes	4
Pick et al. (64)	Prospective cohort	UK	63.3Y	53.9–80.1Y	Urine	983	September 2013–August 2018	PCV13	Yes	8
Benfield et al. (66)	Cross-sectional	Denmark	68Y	58–78Y	Sputum	272	2011	PCV13	Yes	1
Choi et al. (68)	Observational descriptive study and diagnostic accuracy study	South Korea	NR	19–96Y	Sputum/Urine	31	2012–2013	PCV13	No	NA
Harat et al. (69)	Surveillance	Poland	NR	≥50Y	Bronchial aspirates/NP/ Sputum/Urine	77	January 14, 2010–January 13, 2012	PCV13	No	NA
Huijts et al. (70)	Prospective cohort	Netherlands	69Y	57–79Y	Urine	261	January 2008–April 2009	PCV13	Yes	0
Isturiz et al. (71)	Cross-sectional	USA	65Y	18–103Y	Sputum/Urine	585	October 2013–September 2016	PCV13	Yes	6
Prato et al. (73)	Prospective cohort	Italy	79Y	72–83Y	Bronchial aspirates/NP/ Sputum	59	January 2013–January 2015	PCV13	Yes	4
Sando et al. (76)	Prospective cohort	Japan	NR	≥65Y	Sputum	215	2011–2014; May 1, 2016–April 30, 2017	PCV13	Yes	4

* Studies included in the primary post-PHiD-CV/-PCV13 analysis. CAP, community-acquired pneumonia; N, total number of serotyped isolates; n, number of studies; NIP, national immunization program; NP, nasopharyngeal; NR, not reported; PCV, pneumococcal conjugate vaccine; PCV13, 13-valent PCV; PHiD-CV, pneumococcal non-typeable *Haemophilus influenzae* protein D conjugate vaccine; ref, reference; UK, United Kingdom; USA, United States of America; Y, years.

#### 3.3.2 Serotype distribution in adult non-invasive CAP post-PCV13 uptake in infants

**Figure 3:**
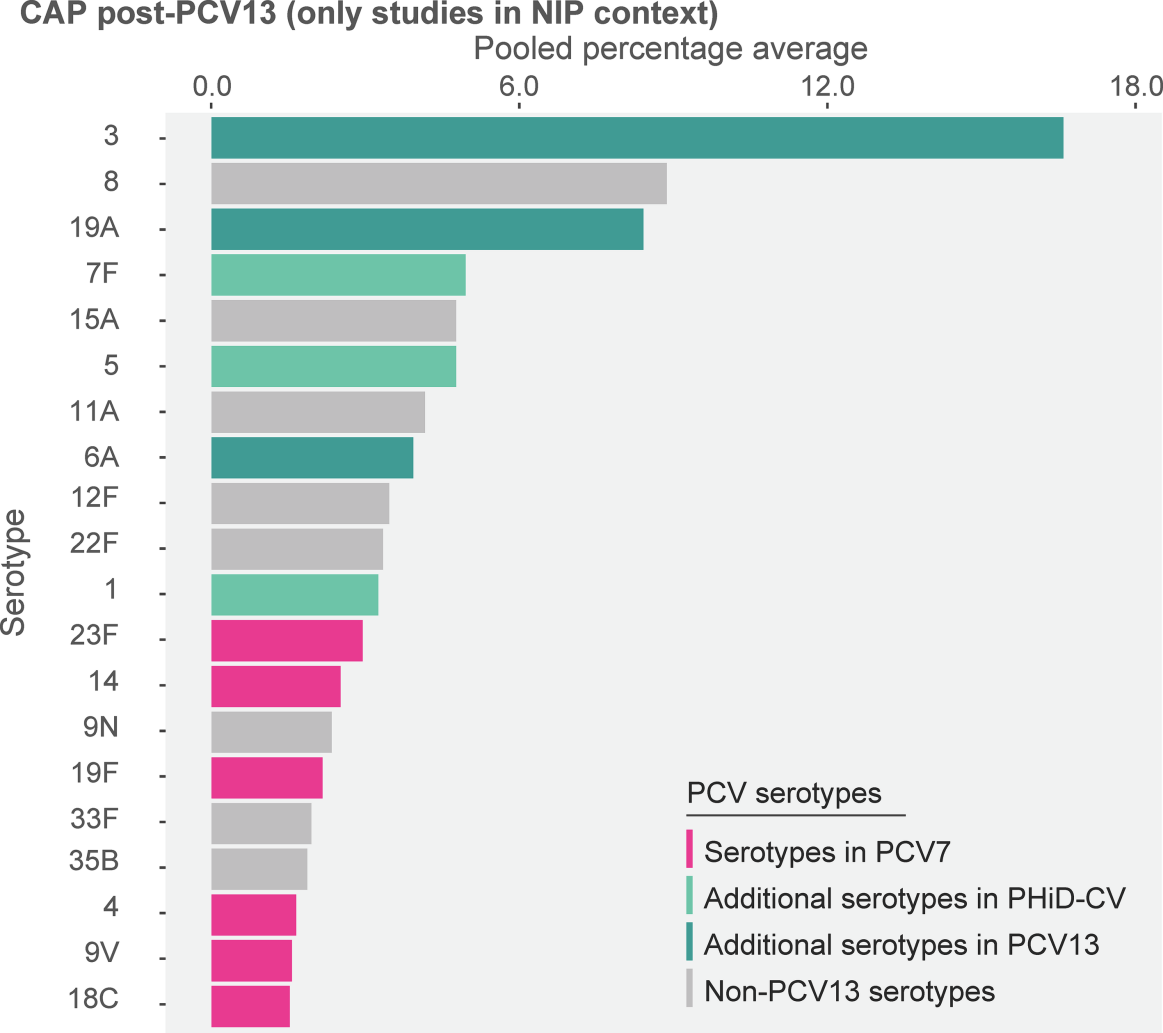
Serotype distribution in non-invasive community-acquired pneumonia among adults ≥65 years post-PCV13 implementation through infant national immunization programs (*n* = 8). The top 20 serotypes are shown. Serotypes are represented by colors corresponding to the lowest valency PCV product in which they are included. In the PCV legend, the additional serotypes included in the product are relative to the next lower valency product. Pooled percentage averages were calculated for each serotype individually, thus the sum of all serotypes may exceed 100%. Serotype-specific pooled percentage averages were calculated only if 5 or more studies reported on the respective serotype. CAP, community-acquired pneumonia; n, number of studies that were included in the analysis; NIP, national immunization program, PCV, pneumococcal conjugate vaccine; PCV7, 7-valent PCV; PCV13, 13-valent PCV; PHiD-CV, pneumococcal non-typeable *Haemophilus influenzae* protein D conjugate vaccine.

Post-PCV13 implementation through infant NIPs (primary analysis comprising data of 8 studies), the 10 most often reported serotypes in older adults with CAP were 3 (16.6%), 8 (8.9%), 19A (8.4%), 7F (4.9%), 15A (4.8%), 5 (4.8%) 11A (4.2%), 6A (3.9%), 12F (3.5%), and 22F (3.3%) (Figure 3; Supplementary Table S4). The sensitivity analysis, which included data of 3 additional studies where PCV13 was marketed only, showed that serotypes 3 (15.2%), 19A (8.4%), and 8 (7.4%) remained top-ranked (Supplementary Figure S4).

In the post-PCV7 era (3 studies, all in infant NIP context), serotype 3 was the leading serotype (16.0%) (Supplementary Figure S5). When comparing the reporting of PCV13 serotypes between both PCV periods, pooled percentage averages were 16.0% post-PCV7 vs. 16.6% post-PHiD-CV/PCV13 for serotype 3, 7.4% vs. 8.4% for 19A, and 3.4% vs. 4.9% for 7F. The reporting of other PCV13 serotypes was low and comparable between both periods (Supplementary Figure S5).

## 4 Discussion

Our analyses show a low reporting rate of most PCV13 serotypes in both IPD and non-invasive CAP among older adults after the implementation of PHiD-CV/PCV13 in infant NIPs. Yet, PCV13-unique serotypes 3 and 19A remained prominent in both manifestations, with pooled percentage averages ranging from 7.1 to 16.6%. Also serotype 7F remained frequently reported (4.4% in IPD and 4.9% in non-invasive CAP). Since most of the included studies were conducted in countries without routine pneumococcal vaccine recommendations for older adults at the time of study conduct [e.g., France (78)] or with low uptake levels among this population [e.g., in many countries in Europe (29) and in Japan (30)], the trends we observed are likely attributed to herd protection by infant PCV use. Because of the limited number of studies in PHiD-CV context, our data primarily show the impact of infant PCV13 use on adult pneumococcal epidemiology.

In line with several other literature reviews/multi-country surveillance studies focusing on pneumococcal epidemiology in adult IPD (18, 21, 79–84) or CAP (85) after infant PHiD-CV/PCV13 implementation, our analyses therefore suggest that herd immunity via infant immunization may not provide sufficient protection in older adults against some serotypes, such as 3 and 19A.

In addition, our analysis identified several frequently reported non-PCV13 serotypes in older adults. Serotype 8 was identified as the second leading serotype in both disease manifestations, with pooled percentage averages of 10.0% (IPD) and 8.9% (CAP). Serotype 22F was another highly ranked non-PCV13 serotype in IPD (8.2%). The identification of these serotypes aligns with the initial results of the Pneumococcal Serotype Replacement and Distribution Estimation (PSERENADE) project collected between 2015 and 2018 (81, 86), an important ongoing, global IPD epidemiology study that uses “raw” surveillance data that may more accurately reflect the current epidemiology and may thus be less prone to bias compared to our study on published observational studies. Other common non-PCV13 serotypes identified in our analyses were 12F and 15A, contributing to both IPD and CAP with a pooled percentage averages ranging from 3.5 to 5.9%. Beyond these shared serotypes, disparities in serotype distribution between IPD and CAP were observed, likely reflecting differences in invasiveness potential of serotypes (87). Several factors may influence the invasiveness potential of serotypes and thus contribute to these differences, including phylogenetic relatedness between serotypes, potential cross-protection between related serotypes, vaccine pressure that is currently mostly focused on IPD-associated serotypes, and antibiotic-selective pressure (88–91). Comprehensive surveillance of both clinical manifestations, ideally incorporating genomic analysis (92), will be crucial to track serotype dynamics and assess the impact of serotype-specific pneumococcal vaccines.

Both serotypes 8 and 22F are included in at least one of the newly licensed vaccines for infants (PCV15/PCV20) (93, 94), thus their anticipated widespread uptake via infant NIPs could lead to a further reduction in pneumococcal disease burden among older adults. Regardless, since herd immunity by infant vaccination may not provide sufficient protection for certain serotypes in adults and the serotype distribution differs between children and adults (86), direct immunization of adults with a vaccine tailored to this population may be a more effective strategy. PCV21, designed based on adult IPD epidemiology, is a prime example of this approach (95). Yet, as our findings indicate that the serotype distribution differs between IPD and non-invasive CAP and given that replacement disease is likely to occur with the use of PCV21, similar to PHiD-CV/PCV13 implementation in infant NIPs (80), the benefit of this vaccine could be temporary (80). In addition, the current PCV technology has been shown to exert carrier-induced epitope suppression, a phenomenon where the immune response to certain serotypes is suppressed due to the presence of carrier proteins (96). As this phenomenon is particularly evident with increasing valency and thus a higher dosage of carrier proteins, the addition of more serotypes to existing PCV formulations may compromise vaccine effectiveness. New vaccine technologies and strategies that can mitigate this phenomenon and thereby broaden and improve protection are therefore essential, targeting serotypes for which current pneumococcal vaccines have been less effective and addressing emerging serotypes across all disease manifestations tailored to older adults.

Our SLR has some limitations. The pooling of data obtained from different settings may have introduced heterogeneity into our findings, which precluded formal statistical comparisons among the different PCV periods and PCV products, such as a meta-analysis. Instead, we calculated a pooled percentage average for each serotype in each analysis, which accounts for the relative importance of values based on the sample size of the corresponding study. The pooled percentage average is grounded in the number of cases for each serotype reported by each study, and thus enhances the robustness and reliability of our findings. However, this may also skew the serotype distribution toward those serotypes with higher reporting rates. Moreover, for our primary analyses, we only included serotypes that were reported by at least 5 studies, aiming to focus on the serotypes with high reporting rates. However, this may have obscured an important emerging serotype reported by a limited number of studies.

In addition, we did not analyze the impact of direct adult vaccination with PPSV23 and/or PCV13 on the serotype distribution, as vaccination rates in older adults were relatively low and uptake data were not consistently available for all the included study periods. Some of the included studies were conducted in settings where pneumococcal vaccination of older adults was recommended (78), although uptake rates were generally low in these regions (29, 30). This limits the differentiation between indirect and direct vaccination effects on the serotype distribution in older adults.

Although a formal risk of bias assessment was not performed, we believe that the rigorous inclusion criteria and critical appraisal of data quality ensured that the included data were adequate for descriptive analyses. However, these strict criteria may have contributed to the inclusion of data that mostly originated from high-income countries, which may limit the generalizability of our findings.

Lastly, our analysis was restricted to data up to 2020 to avoid potential effects of the COVID-19 pandemic on pneumococcal disease epidemiology, including the worldwide decline in pneumococcal disease burden, and disruptions in vaccination programs and surveillance systems (97–100). Consequently, our findings may not fully reflect the current serotype distribution in pneumococcal disease among older adults. However, despite the increased awareness of respiratory infections and the importance of vaccination of older adults due to the COVID-19 pandemic (101, 102), pneumococcal vaccine uptake among older adults has not increased in many countries post-pandemic (103–107). Notably, recent studies indicate that pneumococcal disease due to PCV13 serotypes 3 and 19A has persisted among adults in multiple countries during and following the COVID-19 pandemic, including several European countries, the UK, the US, and Brazil (108–113), supporting the continued relevance of our findings.

In conclusion, the persistent prevalence of specific PCV13 serotypes alongside the concurrent emergence of non-PCV13 serotypes, as well as the differences in epidemiology across the diverse clinical pneumococcal manifestations, present dynamic challenges for optimizing vaccine strategies in older adults. In addition to increasing efforts to improve uptake in this susceptible population, the development of more effective vaccines using improved technologies and broad serotype coverage, will be crucial to enhance the impact on the disease burden in older adults and to address the evolving pneumococcal epidemiology.

## Trademark statement

*Synflorix* is a trademark licensed to or owned by GSK. *Prevenar/Prevnar*, *Prevenar 13/Prevnar 13* and *Prevenar 20/Prevnar 20* are trademarks of Pfizer Inc. *Pneumovax 23*, *Vaxneuvance*, and *Capvaxive* are trademarks of Merck Sharp & Dohme LLC, a subsidiary of Merck & Co., Inc. (MSD).

## References

[1] Lynch JP Zhanel GG. *Streptococcus pneumoniae*: epidemiology, risk factors, and strategies for prevention. Semin Respir Crit Care Med. (2009) 30:189–209. doi: 10.1055/s-0029-1202938, PMID: 19296419

[2] O’Brien KL Wolfson LJ Watt JP Henkle E Deloria-Knoll M McCall N. Burden of disease caused by *Streptococcus pneumoniae* in children younger than 5 years: global estimates. Lancet. (2009) 374:893–902. doi: 10.1016/S0140-6736(09)61204-6, PMID: 19748398

[3] Drijkoningen JJ Rohde GG. Pneumococcal infection in adults: burden of disease. Clin Microbiol Infect. (2014) 20:45–51. doi: 10.1111/1469-0691.12461, PMID: 24313448

[4] Russell F Sanderson C Temple B Global review of the distribution of pneumococcal disease by age and region 2011. Available online at: https://blogs.lshtm.ac.uk/vaccineschedules/files/6.-Russel-review-age-specific-epidemiology-PCV-schedules-session-nov.11.pdf (Accessed April 14, 2025).

[5] Weiser JN. The pneumococcus: why a commensal misbehaves. J Mol Med. (2010) 88:97–102. doi: 10.1007/s00109-009-0557-x, PMID: 19898768 PMC4487619

[6] Ganaie F Saad JS McGee L van Tonder AJ Bentley SD Lo SW. A new pneumococcal capsule type, 10D, is the 100th serotype and has a large cps fragment from an oral Streptococcus. MBio. (2020) 11:11. doi: 10.1128/mBio.00937-20, PMID: 32430472 PMC7240158

[7] World Health Organizaton. Pneumococcal conjugate vaccines in infants and children under 5 years of age: WHO position paper. Wkly Epidemiol Rec. (2019) 94:85–103.

[8] World Health Organization. Pneumococcal conjugate (PCV3): Immunization coverage estimates by World Bank Income Group 2022. Available online at: https://apps.who.int/gho/data/view.main.PCV3vREGWB?lang=en. (Accessed April 14, 2025).

[9] Noharet-Koenig R Lasota K Faivre P Langevin E. Evolution of pneumococcal vaccine recommendations and criteria for decision making in 5 Western European countries and the United States. MDM Policy Pract. (2023) 8:23814683231174432. doi: 10.1177/23814683231174432, PMID: 37274614 PMC10233586

[10] Centers for Disease Control and Prevention. Use of 13-valent pneumococcal conjugate vaccine and 23-valent pneumococcal polysaccharide vaccine among children aged 6-18 years with immunocompromising conditions: recommendations of the advisory committee on immunization practices (ACIP). MMWR Morb Mortal Wkly Rep. (2013) 13:232–5. doi: 10.1111/ajt.12073, PMID: 23803961 PMC4604951

[11] Centers for Disease Control and Prevention. Updated recommendations for prevention of invasive pneumococcal disease among adults using the 23-valent pneumococcal polysaccharide vaccine (PPSV23). MMWR Morb Mortal Wkly Rep. (2010) 59:1102–6. 20814406

[12] Bonnave C Mertens D Peetermans W Cobbaert K Ghesquiere B Deschodt M. Adult vaccination for pneumococcal disease: a comparison of the national guidelines in Europe. Eur J Clin Microbiol Infect Dis. (2019) 38:785–91. doi: 10.1007/s10096-019-03485-3, PMID: 30778705

[13] Sings HL Gessner BD Wasserman MD Jodar L. Pneumococcal conjugate vaccine impact on serotype 3: a review of surveillance data. Infect Dis Ther. (2021) 10:521–39. doi: 10.1007/s40121-021-00406-w, PMID: 33587245 PMC7954992

[14] Kobayashi M Pilishvili T Farrar JL Leidner AJ Gierke R Prasad N. Pneumococcal vaccine for adults aged ≥19 years: recommendations of the advisory committee on immunization practices, United States, 2023. MMWR Recomm Rep. (2023) 72:1–39. doi: 10.15585/mmwr.rr7203a1, PMID: 37669242 PMC10495181

[15] Juergens C de Villiers PJ Moodley K Jayawardene D Jansen KU Scott DA. Safety and immunogenicity of 13-valent pneumococcal conjugate vaccine formulations with and without aluminum phosphate and comparison of the formulation of choice with 23-valent pneumococcal polysaccharide vaccine in elderly adults: a randomized open-label trial. Hum Vaccin Immunother. (2014) 10:1343–53. doi: 10.4161/hv.27998, PMID: 24576885 PMC4896606

[16] Falkenhorst G Remschmidt C Harder T Hummers-Pradier E Wichmann O Bogdan C. Effectiveness of the 23-valent pneumococcal polysaccharide vaccine (PPV23) against pneumococcal disease in the elderly: systematic review and Meta-analysis. PLoS One. (2017) 12:e0169368. doi: 10.1371/journal.pone.0169368, PMID: 28061505 PMC5218810

[17] Kobayashi M Farrar JL Gierke R Britton A Childs L Leidner AJ. Use of 15-valent pneumococcal conjugate vaccine and 20-valent pneumococcal conjugate vaccine among U.S. adults: updated recommendations of the advisory committee on immunization practices-United States, 2022. MMWR Morb Mortal Wkly Rep. (2022) 71:109–17. doi: 10.15585/mmwr.mm7104a1 35085226 PMC9351524

[18] Cohen O Knoll MD O’Brien KL Ramakrishnan M Farrar J Pilishvili T. Pneumococcal conjugate vaccine (PCV) review of impact evidence (PRIME): summary of findings from systematic review. World Health Organization (2017). Available online at: https://terrance.who.int/mediacentre/data/sage/SAGE_Docs_Ppt_Oct2017/9_session_PCV/Oct2019_session9_PCV_PRIMEsummary.pdf (Accessed April 14, 2025).

[19] Cohen R Cohen JF Chalumeau M Levy C. Impact of pneumococcal conjugate vaccines for children in high-and non-high-income countries. Expert Rev Vaccines. (2017) 16:625–40. doi: 10.1080/14760584.2017.1320221, PMID: 28409537

[20] Wahl B O’Brien KL Greenbaum A Majumder A Liu L Chu Y. Burden of Streptococcus pneumoniae and *Haemophilus influenzae* type b disease in children in the era of conjugate vaccines: global, regional, and national estimates for 2000-15. Lancet Glob Health. (2018) 6:e744–57. doi: 10.1016/s2214-109x(18)30247-x, PMID: 29903376 PMC6005122

[21] Izurieta P Bahety P Adegbola R Clarke C Hoet B. Public health impact of pneumococcal conjugate vaccine infant immunization programs: assessment of invasive pneumococcal disease burden and serotype distribution. Expert Rev Vaccines. (2018) 17:479–93. doi: 10.1080/14760584.2018.1413354, PMID: 29241390

[22] Ahmed SS Pondo T Xing W McGee L Farley M Schaffner W. Early impact of 13-valent pneumococcal conjugate vaccine use on invasive pneumococcal disease among adults with and without underlying medical conditions-United States. Clin Infect Dis. (2020) 70:2484–92. doi: 10.1093/cid/ciz739, PMID: 31402387

[23] Vadlamudi NK Chen A Marra F. Impact of the 13-valent pneumococcal conjugate vaccine among adults: a systematic review and Meta-analysis. Clin Infect Dis. (2019) 69:34–49. doi: 10.1093/cid/ciy872, PMID: 30312379

[24] Van Buynder P Booy R. Pneumococcal vaccination in older persons: where are we today? Pneumonia. (2018) 10:1. doi: 10.1186/s41479-017-0045-y, PMID: 29318106 PMC5755353

[25] Latifi-Navid H Latifi-Navid S Mostafaiy B Jamalkandi SA Ahmadi A. Pneumococcal disease and the effectiveness of the PPV23 vaccine in adults: a two-stage Bayesian Meta-analysis of observational and RCT reports. Sci Rep. (2018) 8:11051. doi: 10.1038/s41598-018-29280-2, PMID: 30038423 PMC6056566

[26] See KC. Pneumococcal vaccination in adults: a narrative review of considerations for individualized decision-making. Vaccines. (2023) 11:11. doi: 10.3390/vaccines11050908, PMID: 37243012 PMC10223523

[27] Centers for Disease Control and Prevention. Vaccination coverage among adults in the United States, national health interview survey, 2019–2020. Available online at: https://www.cdc.gov/adultvaxview/publications-resources/vaccination-coverage-adults-2019-2020.html (Accessed April 14, 2025).

[28] Government of Canada. Vaccine uptake in Canadian adults 2019. Available online at: https://www.canada.ca/en/public-health/services/publications/healthy-living/2018-2019-influenza-flu-vaccine-coverage-survey-results.html. (Accessed April 14, 2025).

[29] Ipsos. Adult pneumonia vaccination understanding in Europe: 65 years and over. Available onlien at: https://www.ipsos.com/sites/default/files/ct/publication/documents/2017-10/ipsos-healthcare-pneu-vue-65s-and-over-report_0.pdf (Accessed April 14, 2025).

[30] Yamada N Nakatsuka K Tezuka M Murata F Maeda M Akisue T. Pneumococcal vaccination coverage and vaccination-related factors among older adults in Japan: LIFE study. Vaccine. (2024) 42:239–45. doi: 10.1016/j.vaccine.2023.12.009, PMID: 38087713

[31] Weinberger DM Malley R Lipsitch M. Serotype replacement in disease after pneumococcal vaccination. Lancet. (2011) 378:1962–73. doi: 10.1016/S0140-6736(10)62225-8, PMID: 21492929 PMC3256741

[32] Hausdorff WP Bryant J Paradiso PR Siber GR. Which pneumococcal serogroups cause the most invasive disease: implications for conjugate vaccine formulation and use, part I. Clin Infect Dis. (2000) 30:100–21. doi: 10.1086/313608, PMID: 10619740

[33] Hausdorff WP Bryant J Kloek C Paradiso PR Siber GR. The contribution of specific pneumococcal serogroups to different disease manifestations: implications for conjugate vaccine formulation and use, part II. Clin Infect Dis. (2000) 30:122–40. doi: 10.1086/313609, PMID: 10619741

[34] Hausdorff WP Feikin DR Klugman KP. Epidemiological differences among pneumococcal serotypes. Lancet Infect Dis. (2005) 5:83–93. doi: 10.1016/s1473-3099(05)01280-6, PMID: 15680778

[35] Song JY Nahm MH Moseley MA. Clinical implications of pneumococcal serotypes: invasive disease potential, clinical presentations, and antibiotic resistance. J Korean Med Sci. (2013) 28:4–15. doi: 10.3346/jkms.2013.28.1.4, PMID: 23341706 PMC3546102

[36] Earle K Williams S. Burden of pneumococcal disease in adults aged 65 years and older: an Australian perspective. Pneumonia. (2016) 8:9. doi: 10.1186/s41479-016-0008-8, PMID: 28702288 PMC5471924

[37] Musher DM Montoya R Wanahita A. Diagnostic value of microscopic examination of gram-stained sputum and sputum cultures in patients with bacteremic pneumococcal pneumonia. Clin Infect Dis. (2004) 39:165–9. doi: 10.1086/421497, PMID: 15307023

[38] Pride MW Huijts SM Wu K Souza V Passador S Tinder C. Validation of an immunodiagnostic assay for detection of 13 *Streptococcus pneumoniae* serotype-specific polysaccharides in human urine. Clin Vaccine Immunol. (2012) 19:1131–41. doi: 10.1128/cvi.00064-12, PMID: 22675155 PMC3416073

[39] Kalina WV Souza V Wu K Giardina P McKeen A Jiang Q. Qualification and clinical validation of an immunodiagnostic assay for detecting 11 additional *Streptococcus pneumoniae* serotype–specific polysaccharides in human urine. Clin Infect Dis. (2020) 71:e430–8. doi: 10.1093/cid/ciaa158, PMID: 32072165 PMC7713672

[40] Sheppard CL Harrison TG Smith MD George RC. Development of a sensitive, multiplexed immunoassay using xMAP beads for detection of serotype-specific *streptococcus pneumoniae* antigen in urine samples. J Med Microbiol. (2011) 60:49–55. doi: 10.1099/jmm.0.023150-0, PMID: 20864547

[41] Izurieta P AbdelGhany M Borys D. Serotype distribution of invasive and non-invasive pneumococcal disease in children ≤5 years of age following the introduction of 10-and 13-valent pneumococcal conjugate vaccines in infant national immunization programs: a systematic literature review. Frontiers in Public Health (2025) 13. doi: 10.3389/fpubh.2025.1544359 PMC12162640 40520299

[42] Page MJ McKenzie JE Bossuyt PM Boutron I Hoffmann TC Mulrow CD. The PRISMA 2020 statement: an updated guideline for reporting systematic reviews. BMJ. (2021) 372:n71. doi: 10.1136/bmj.n71, PMID: 33782057 PMC8005924

[43] National Institute for Health and Care Excellence. Pneumonia: diagnosis and management of community-and hospital-acquired pneumonia in adults (2012). Available online at: https://www.nice.org.uk/guidance/cg191/documents/pneumonia-final-scope2 (Accessed April 14, 2025). 25520986

[44] Randle E Ninis N Inwald D. Invasive pneumococcal disease. Arch Dis Child Educ Pract Ed. (2011) 96:183–90. doi: 10.1136/adc.2010.191718, PMID: 21555595

[45] Amin-Chowdhury Z Collins S Sheppard C Litt D Fry NK Andrews N. Characteristics of invasive pneumococcal disease caused by emerging serotypes after the introduction of the 13-valent pneumococcal conjugate vaccine in England: a prospective observational cohort study, 2014-2018. Clin Infect Dis. (2020) 71:e235–43. doi: 10.1093/cid/ciaa043, PMID: 31955196

[46] Ciruela P Broner S Izquierdo C Pallares R Munoz-Almagro C Hernandez S. Indirect effects of paediatric conjugate vaccines on invasive pneumococcal disease in older adults. Int J Infect Dis. (2019) 86:122–30. doi: 10.1016/j.ijid.2019.06.030, PMID: 31283992

[47] Danis K Varon E Lepoutre A Janssen C Forestier E Epaulard O. Factors associated with severe nonmeningitis invasive pneumococcal disease in adults in France. Open forum. Infect Dis. (2019) 6:ofz510. doi: 10.1093/ofid/ofz510, PMID: 31868865 PMC6918451

[48] de Miguel S Domenech M Gonzalez-Camacho F Sempere J Vicioso D Sanz JC. Nationwide trends of invasive pneumococcal disease in Spain from 2009 through 2019 in children and adults during the pneumococcal conjugate vaccine era. Clin Infect Dis. (2021) 73:e3778–87. doi: 10.1093/cid/ciaa1483, PMID: 32990303

[49] Kim SH Chung DR Song JH Baek JY Thamlikitkul V Wang H. Changes in serotype distribution and antimicrobial resistance of *Streptococcus pneumoniae* isolates from adult patients in Asia: emergence of drug-resistant non-vaccine serotypes. Vaccine. (2020) 38:6065–73. doi: 10.1016/j.vaccine.2019.09.065, PMID: 31590932

[50] Ladhani SN Collins S Djennad A Sheppard CL Borrow R Fry NK. Rapid increase in non-vaccine serotypes causing invasive pneumococcal disease in England and Wales, 2000-17: a prospective national observational cohort study. Lancet Infect Dis. (2018) 18:441–51. doi: 10.1016/S1473-3099(18)30052-5, PMID: 29395999

[51] Li MC Wang Y Zhang H Liu Y Chen XJ Yang HW. Serotype distribution and clinical characteristics associated with *streptococcus pneumoniae* among Chinese children and adults with invasive pneumococcal disease: a multicenter observational study. Hum Vaccin Immunother. (2021) 17:146–56. doi: 10.1080/21645515.2020.1757996, PMID: 32530720 PMC7872053

[52] Ouldali N Varon E Levy C Angoulvant F Georges S Ploy MC. Invasive pneumococcal disease incidence in children and adults in France during the pneumococcal conjugate vaccine era: an interrupted time-series analysis of data from a 17-year national prospective surveillance study. Lancet Infect Dis. (2021) 21:137–47. doi: 10.1016/S1473-3099(20)30165-1, PMID: 32702302

[53] Park DC Kim SH Yong D Suh IB Kim YR Yi J. Serotype distribution and antimicrobial resistance of invasive and noninvasive *Streptococcus pneumoniae* isolates in Korea between 2014 and 2016. Ann Lab Med. (2019) 39:537–44. doi: 10.3343/alm.2019.39.6.537, PMID: 31240881 PMC6660335

[54] Ubukata K Takata M Morozumi M Chiba N Wajima T Hanada S. Effects of pneumococcal conjugate vaccine on genotypic penicillin resistance and serotype changes, Japan, 2010-2017. Emerg Infect Dis. (2018) 24:2010–20. doi: 10.3201/eid2411.180326, PMID: 30334707 PMC6200004

[55] Yanagihara K Kosai K Mikamo H Mukae H Takesue Y Abe M. Serotype distribution and antimicrobial susceptibility of *Streptococcus pneumoniae* associated with invasive pneumococcal disease among adults in Japan. Int J Infect Dis. (2021) 102:260–8. doi: 10.1016/j.ijid.2020.10.017, PMID: 33065297

[56] Zintgraff J Fossati S Pereira CS Veliz O Regueira M Moscoloni MA. Distribution of PCV13 and PPSV23 *Streptococcus pneumoniae* serotypes in Argentinean adults with invasive disease, 2013-2017. Rev Argent Microbiol. (2020) 52:189–94. doi: 10.1016/j.ram.2019.11.004, PMID: 31932112

[57] Lee HY Wu TL Su LH Li HC Janapatla RP Chen CL. Invasive pneumococcal disease caused by ceftriaxone-resistant *Streptococcus pneumoniae* in Taiwan. J Microbiol Immunol Infect. (2018) 51:500–9. doi: 10.1016/j.jmii.2016.12.004, PMID: 28690025

[58] Ciruela P Izquierdo C Broner S Munoz-Almagro C Hernandez S Ardanuy C. The changing epidemiology of invasive pneumococcal disease after PCV13 vaccination in a country with intermediate vaccination coverage. Vaccine. (2018) 36:7744–52. doi: 10.1016/j.vaccine.2018.05.026, PMID: 30473132

[59] Richter L Schmid D Kanitz EE Zwazl I Pollabauer E Jasinska J. Invasive pneumococcal diseases in children and adults before and after introduction of the 10-valent pneumococcal conjugate vaccine into the Austrian national immunization program. PLoS One. (2019) 14:e0210081. doi: 10.1371/journal.pone.0210081, PMID: 30629620 PMC6328268

[60] Fenoll A Ardanuy C Linares J Cercenado E Marco F Fleites A. Serotypes and genotypes of *S. pneumoniae* isolates from adult invasive disease in Spain: a 5-year prospective surveillance after pediatric PCV13 licensure. The ODIN study. Vaccine. (2018) 36:7993–8000. doi: 10.1016/j.vaccine.2018.10.098, PMID: 30449634

[61] Demczuk WHB Martin I Desai S Griffith A Caron-Poulin L Lefebvre B. Serotype distribution of invasive *Streptococcus pneumoniae* in adults 65 years of age and over after the introduction of childhood 13-valent pneumococcal conjugate vaccination programs in Canada, 2010-2016. Vaccine. (2018) 36:4701–7. doi: 10.1016/j.vaccine.2018.06.018, PMID: 29937245

[62] Forstner C Kolditz M Kesselmeier M Ewig S Rohde G Barten-Neiner G. Pneumococcal conjugate serotype distribution and predominating role of serotype 3 in German adults with community-acquired pneumonia. Vaccine. (2020) 38:1129–36. doi: 10.1016/j.vaccine.2019.11.026, PMID: 31761500

[63] LeBlanc J ElSherif M Ye L Mac Kinnon-Cameron D Ambrose A Hatchette TF. Age-stratified burden of pneumococcal community acquired pneumonia in hospitalised Canadian adults from 2010 to 2015. BMJ Open Respir Res. 7:7. doi: 10.1136/bmjresp-2019-000550, PMID: 32188585 PMC7078693

[64] Pick H Daniel P Rodrigo C Bewick T Ashton D Lawrence H. Pneumococcal serotype trends, surveillance and risk factors in UK adult pneumonia, 2013-18. Thorax. (2020) 75:38–49. doi: 10.1136/thoraxjnl-2019-213725, PMID: 31594801

[65] Sherwin RL Gray S Alexander R McGovern PC Graepel J Pride MW. Distribution of 13-valent pneumococcal conjugate vaccine *Streptococcus pneumoniae* serotypes in US adults aged >/=50 years with community-acquired pneumonia. J Infect Dis. (2013) 208:1813–20. doi: 10.1093/infdis/jit506, PMID: 24092845

[66] Benfield T Skovgaard M Schonheyder HC Knudsen JD Bangsborg J Ostergaard C. Serotype distribution in non-bacteremic pneumococcal pneumonia: association with disease severity and implications for pneumococcal conjugate vaccines. PLoS One. (2013) 8:e72743. doi: 10.1371/journal.pone.0072743, PMID: 24009703 PMC3751823

[67] Bewick T Sheppard C Greenwood S Slack M Trotter C George R. Serotypes associated with the development of pneumococcal Para-pneumonic effusion in adults. Eur Respir J. (2013) 42:733–41. doi: 10.1183/09031936.00144712, PMID: 23258784

[68] Choi MJ Song JY Cheong HJ Jeon JH Kang SH Jung EJ. Clinical usefulness of pneumococcal urinary antigen test, stratified by disease severity and serotypes. J Infect Chemother. (2015) 21:672–9. doi: 10.1016/j.jiac.2015.06.003, PMID: 26212866 PMC7128949

[69] Harat R Alexander R Gray S Gutterman EM Pluta J Pride M. Prospective, population-based surveillance of the burden of *Streptococcus pneumoniae* in community-acquired pneumonia in older adults, Chrzanow County, Poland, 2010 to 2012. Pneumonol Alergol Pol. (2016) 84:95–103. doi: 10.5603/PiAP.2016.0007, PMID: 27238167

[70] Huijts SM Pride MW Vos JM Jansen KU Webber C Gruber W. Diagnostic accuracy of a serotype-specific antigen test in community-acquired pneumonia. Eur Respir J. (2013) 42:1283–90. doi: 10.1183/09031936.00137412, PMID: 23397295

[71] Isturiz RE Ramirez J Self WH Grijalva CG Counselman FL Volturo G. Pneumococcal epidemiology among us adults hospitalized for community-acquired pneumonia. Vaccine. (2019) 37:3352–61. doi: 10.1016/j.vaccine.2019.04.087, PMID: 31072732

[72] Morimoto K Suzuki M Ishifuji T Yaegashi M Asoh N Hamashige N. The burden and etiology of community-onset pneumonia in the aging Japanese population: a multicenter prospective study. PLoS One. (2015) 10:e0122247. doi: 10.1371/journal.pone.0122247, PMID: 25822890 PMC4378946

[73] Prato R Fortunato F Cappelli MG Chironna M Martinelli D. Effectiveness of the 13-valent pneumococcal conjugate vaccine against adult pneumonia in Italy: a case-control study in a 2-year prospective cohort. BMJ Open. (2018) 8:e019034. doi: 10.1136/bmjopen-2017-019034, PMID: 29581200 PMC5875676

[74] Rodrigo C Bewick T Sheppard C Greenwood S Macgregor V Trotter C. Pneumococcal serotypes in adult non-invasive and invasive pneumonia in relation to child contact and child vaccination status. Thorax. (2014) 69:168–73. doi: 10.1136/thoraxjnl-2013-203987, PMID: 24048505

[75] Rodrigo C Bewick T Sheppard C Greenwood S McKeever TM Trotter CL. Impact of infant 13-valent pneumococcal conjugate vaccine on serotypes in adult pneumonia. Eur Respir J. (2015) 45:1632–41. doi: 10.1183/09031936.00183614, PMID: 25792633

[76] Sando E Suzuki M Furumoto A Asoh N Yaegashi M Aoshima M. Impact of the pediatric 13-valent pneumococcal conjugate vaccine on serotype distribution and clinical characteristics of pneumococcal pneumonia in adults: the Japan pneumococcal vaccine effectiveness study (J-PAVE). Vaccine. (2019) 37:2687–93. doi: 10.1016/j.vaccine.2019.04.009, PMID: 30975569

[77] Suzuki M Dhoubhadel BG Ishifuji T Yasunami M Yaegashi M Asoh N. Serotype-specific effectiveness of 23-valent pneumococcal polysaccharide vaccine against pneumococcal pneumonia in adults aged 65 years or older: a multicentre, prospective, test-negative design study. Lancet Infect Dis. (2017) 17:313–21. doi: 10.1016/S1473-3099(17)30049-X, PMID: 28126327

[78] Farge G de Wazières B Raude J Delavelle C Humbert F Janssen C. The health Professional’s view on the inclusion of age in the recommendations for pneumococcal vaccination: results of a cross-sectional survey in France. Geriatrics. (2021) 7:4. doi: 10.3390/geriatrics7010004, PMID: 35076506 PMC8788281

[79] Hanquet G Krizova P Valentiner-Branth P Ladhani SN Nuorti JP Lepoutre A. Effect of childhood pneumococcal conjugate vaccination on invasive disease in older adults of 10 European countries: implications for adult vaccination. Thorax. (2019) 74:473–82. doi: 10.1136/thoraxjnl-2018-211767 30355641 PMC6484683

[80] Hanquet G Krizova P Dalby T Ladhani SN Nuorti JP Danis K. Serotype replacement after introduction of 10-valent and 13-valent pneumococcal conjugate vaccines in 10 countries. Europe Emerg Infect Dis. (2022) 28:137–8. doi: 10.3201/eid2801.210734, PMID: 34932457 PMC8714201

[81] Garcia Quesada M Yang Y Bennett JC Hayford K Zeger SL Feikin DR. Serotype distribution of remaining pneumococcal meningitis in the mature PCV10/13 period: findings from the PSERENADE project. Microorganisms. (2021) 9:9. doi: 10.3390/microorganisms9040738, PMID: 33916227 PMC8066874

[82] Grant LR Slack MPE Theilacker C Vojicic J Dion S Reinert R-R. Distribution of serotypes causing invasive pneumococcal disease in children from high-income countries and the impact of pediatric pneumococcal vaccination. Clin Infect Dis. (2022) 76:e1062–70. doi: 10.1093/cid/ciac475, PMID: 35789262 PMC9907512

[83] Palmborg A Skovdal M Molden T Åhman H Chen L Banefelt J. Invasive pneumococcal disease among the elderly in the later era of paediatric pneumococcal conjugate vaccination-a longitudinal study over 10 years based on public surveillance data in the Nordics. PLoS One. (2023) 18:e0287378. doi: 10.1371/journal.pone.0287378, PMID: 37363884 PMC10292715

[84] Teixeira R Kossyvaki V Galvez P Méndez C. Pneumococcal serotype evolution and burden in European adults in the last decade: a systematic review. Microorganisms. (2023) 11:11. doi: 10.3390/microorganisms11061376, PMID: 37374878 PMC10303156

[85] Lansbury L Lim B McKeever TM Lawrence H Lim WS. Non-invasive pneumococcal pneumonia due to vaccine serotypes: a systematic review and meta-analysis. eClinicalMedicine. (2022) 44:101271. doi: 10.1016/j.eclinm.2022.101271, PMID: 35112072 PMC8790487

[86] Garcia Quesada M Peterson ME Bennett JC Hayford K Zeger SL Yang Y. Serotype distribution of remaining invasive pneumococcal disease after extensive use of ten-valent and 13-valent pneumococcal conjugate vaccines (the PSERENADE project): a global surveillance analysis. Lancet Infect Dis. (2024) 25:445–56. doi: 10.1016/s1473-3099(24)00588-7, PMID: 39706205 PMC11947070

[87] Balsells E Dagan R Yildirim I Gounder PP Steens A Muñoz-Almagro C. The relative invasive disease potential of *Streptococcus pneumoniae* among children after PCV introduction: a systematic review and meta-analysis. J Infect. (2018) 77:368–78. doi: 10.1016/j.jinf.2018.06.004, PMID: 29964140

[88] Cremers AJ Mobegi FM de Jonge MI van Hijum SA Meis JF Hermans PW. The post-vaccine microevolution of invasive *Streptococcus pneumoniae*. Sci Rep. (2015) 5:14952. doi: 10.1038/srep14952, PMID: 26492862 PMC4615977

[89] Varghese R Neeravi A Subramanian N Pavithra B Kavipriya A Kumar JL. Clonal similarities and sequence-type diversity of invasive and carriage *Streptococcus pneumoniae* in India among children under 5 years. Indian J Med Microbiol. (2019) 37:358–61. doi: 10.4103/ijmm.IJMM_19_348, PMID: 32003333

[90] Gladstone RA Lo SW Lees JA Croucher NJ van Tonder AJ Corander J. International genomic definition of pneumococcal lineages, to contextualise disease, antibiotic resistance and vaccine impact. EBioMedicine. (2019) 43:338–46. doi: 10.1016/j.ebiom.2019.04.021, PMID: 31003929 PMC6557916

[91] Feemster K Hausdorff WP Banniettis N Platt H Velentgas P Esteves-Jaramillo A. Implications of cross-reactivity and cross-protection for pneumococcal vaccine development. Vaccines. (2024) 12:12. doi: 10.3390/vaccines12090974, PMID: 39340006 PMC11435891

[92] Global Pneumococcal Sequencing Project. Available online at: https://www.pneumogen.net/gps/#/about (Accessed April 14, 2025).

[93] U.S. Food and Drug Administration. VAXNEUVANCE Package insert. (2021). Available online at: https://www.fda.gov/vaccines-blood-biologics/vaccines/vaxneuvance (Accessed April 14, 2025).

[94] U.S. Food and Drug Administration. PREVNAR 20 Package insert. (2021). Available online at: https://www.fda.gov/vaccines-blood-biologics/vaccines/prevnar-20 (Accessed April 14, 2025).

[95] Wateska AR Nowalk MP Lin CJ Harrison LH Schaffner W Zimmerman RK. Cost-effectiveness of an in-development adult-formulated pneumococcal vaccine in older US adults. Vaccine. (2023) 41:4431–7. doi: 10.1016/j.vaccine.2023.06.007, PMID: 37316409 PMC10330932

[96] Dagan R Poolman J Siegrist CA. Glycoconjugate vaccines and immune interference: a review. Vaccine. (2010) 28:5513–23. doi: 10.1016/j.vaccine.2010.06.026, PMID: 20600514

[97] Shaw D Abad R Amin-Chowdhury Z Bautista A Bennett D Broughton K. Trends in invasive bacterial diseases during the first 2 years of the COVID-19 pandemic: analyses of prospective surveillance data from 30 countries and territories in the IRIS consortium. Lancet Digit Health. (2023) 5:e582–93. doi: 10.1016/s2589-7500(23)00108-5, PMID: 37516557 PMC10914672

[98] Ota MOC Badur S Romano-Mazzotti L Friedland LR. Impact of COVID-19 pandemic on routine immunization. Ann Med. (2021) 53:2286–97. doi: 10.1080/07853890.2021.2009128, PMID: 34854789 PMC8648038

[99] Principi N Autore G Ramundo G Esposito S. Epidemiology of respiratory infections during the COVID-19 pandemic. Viruses. (2023) 15:15. doi: 10.3390/v15051160, PMID: 37243246 PMC10224029

[100] Bigouette JP Callaghan AW Donadel M Porter AM Rosencrans L Lickness JS. Effects of COVID-19 on vaccine-preventable disease surveillance Systems in the World Health Organization African Region, 2020. Emerg Infect Dis. (2022) 28:S203–7. doi: 10.3201/eid2813.220088, PMID: 36502406 PMC9745246

[101] Zhu J Cole CB Fihman J Adjagba A Dasic M Cernuschi T. Opportunities to accelerate immunization progress in middle-income countries. Vaccine. (2024) 42:S98–s106. doi: 10.1016/j.vaccine.2023.06.079, PMID: 37460357

[102] Kirubarajan A Lynch M Nasreen S Gebretekle GB Fadel SA Crowcroft NS. Increasing pneumococcal vaccine uptake in older adults: a scoping review of interventions in high-income countries. BMC Geriatr. (2023) 23:2. doi: 10.1186/s12877-022-03653-9, PMID: 36593474 PMC9807101

[103] Janssens A Vaes B Abels C Crèvecoeur J Mamouris P Merckx B. Pneumococcal vaccination coverage and adherence to recommended dosing schedules in adults: a repeated cross-sectional study of the INTEGO morbidity registry. BMC Public Health. (2023) 23:1104. doi: 10.1186/s12889-023-15939-7, PMID: 37286969 PMC10245355

[104] Novaes J de Freitas Faria FM de Bragança BSC Dos Santos LI. Impacts of the COVID-19 pandemic on immunization with pneumococcal vaccines in children and older adults in Brazil. Prev Med. (2023) 173:107602. doi: 10.1016/j.ypmed.2023.107602, PMID: 37423475

[105] Lan C Chen YC Chang YI Chuang PC. Impact of COVID-19 outbreak on influenza and pneumococcal vaccination uptake: a multi-center retrospective study. Vaccines. (2023) 11:11. doi: 10.3390/vaccines11050986, PMID: 37243090 PMC10223787

[106] Eiden AL DiFranzo A Bhatti A Echo Wang H Bencina G Yao L. Changes in vaccine administration trends across the life-course during the COVID-19 pandemic in the United States: a claims database study. Expert Rev Vaccines. (2023) 22:481–94. doi: 10.1080/14760584.2023.2217257, PMID: 37218717

[107] Chan PS Poon J Han SC Ye D Yu FY Fang Y. Changes in the pneumococcal vaccination uptake and its determinants before, during, and after the COVID-19 pandemic among community-living older adults in Hong Kong, China. Vaccines. (2024) 12:12. doi: 10.3390/vaccines12080894, PMID: 39204020 PMC11359888

[108] Casanova C Küffer M Leib SL Hilty M. Re-emergence of invasive pneumococcal disease (IPD) and increase of serotype 23B after easing of COVID-19 measures, Switzerland, 2021. Emerg Microbes Infect. (2021) 10:2202–4. doi: 10.1080/22221751.2021.2000892, PMID: 34723783 PMC8648035

[109] Calvo-Silveria S González-Díaz A Marimón JM Cercenado E Quesada MD Casabella A. Resilience and emergence of pneumococcal serotypes and lineages in adults post-PCV13 in Spain: a multicentre study. J Infect Public Health. (2025) 18:102619. doi: 10.1016/j.jiph.2024.102619, PMID: 39662160

[110] Perniciaro S van der Linden M Weinberger DM. Reemergence of invasive pneumococcal disease in Germany during the spring and summer of 2021. Clin Infect Dis. (2022) 75:1149–53. doi: 10.1093/cid/ciac100, PMID: 35136983 PMC9383454

[111] Hyams C Challen R Hettle D Amin-Chowdhury Z Grimes C Ruffino G. Serotype distribution and disease severity in adults hospitalized with *Streptococcus pneumoniae* infection, Bristol and Bath, UK, 2006–2022. Emerg Infect Dis. (2023) 29:1953–64. doi: 10.3201/eid2910.230519, PMID: 37735739 PMC10521591

[112] Ramirez JA Hubler RA Ali M Gray SL Carrico R McNaughton CD. *Streptococcus pneumoniae* serotype distribution among US adults hospitalized with community-acquired pneumonia, 2019-2020. Open Forum Infect Dis. (2025) 12:ofae 727. doi: 10.1093/ofid/ofae727, PMID: 39758745 PMC11697090

[113] Almeida SCG Lemos APS Bierrenbach AL Moraes JC Brandileone MCC. Serotype distribution and antimicrobial susceptibility pattern of *Streptococcus pneumoniae* in COVID-19 pandemic era in Brazil. Microorganisms. (2024) 12:12. doi: 10.3390/microorganisms12020401, PMID: 38399805 PMC10893029

