# Supplementary material for: Serotype distribution of invasive and non-invasive pneumococcal disease in adults ≥65 years of age following the introduction of 10- and 13-valent pneumococcal conjugate vaccines in infant national immunization programs: a systematic literature review
Source: Front Public Health. 2025 May 30;13:1544331. doi: 10.3389/fpubh.2025.1544331 (PMC12162951; doi:10.3389/fpubh.2025.1544331)
Supplement: Supplementary file 1 [file Data_Sheet_1.docx]

Supplementary Material

# Supplementary methods

## Detailed search strategies

PubMed Search Strategy

1. (serotype[Title/Abstract]) AND (((PCV[Title/Abstract]) OR (PHiD-CV[Title/Abstract]) OR (10-valent[Title/Abstract]) OR (PCV10[Title/Abstract]) OR (13-valent[Title/Abstract]) OR (PCV13[Title/Abstract])) OR ((pneumonia[Title/Abstract]) OR (IPD[Title/Abstract]) OR (Otitis[Title/Abstract]) OR (invasive pneumococcal disease[Title/Abstract])))
2. ((etiology[Title/Abstract]) AND (Streptococcus pneumoniae[Title/Abstract])) AND (((PCV[Title/Abstract]) OR (PHiD-CV[Title/Abstract]) OR (10-valent[Title/Abstract]) OR (PCV10[Title/Abstract]) OR (13-valent[Title/Abstract]) OR (PCV13[Title/Abstract])) OR ((pneumonia[Title/Abstract]) OR (IPD[Title/Abstract]) OR (Otitis[Title/Abstract]) OR (invasive pneumococcal disease[Title/Abstract])))

Embase search strategy


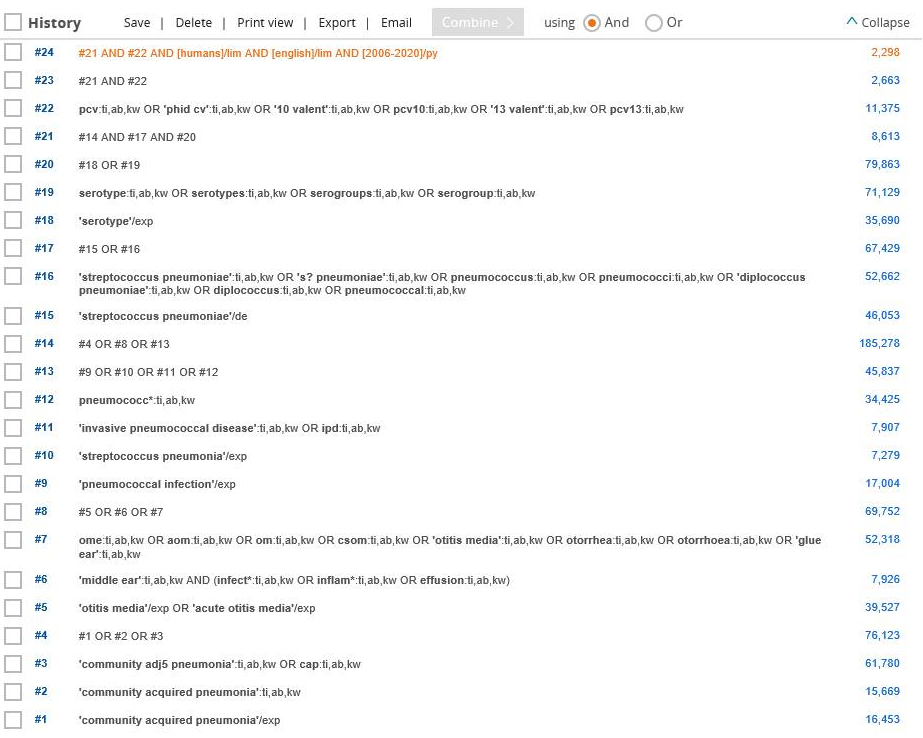


## List of inclusion and exclusion criteria

**Table S1.** IPD eligibility criteria

|  | **Inclusion** | **Exclusion** |
| --- | --- | --- |
| **Population** | - IPD^#^ in adults ≥65 years   - The study population (hospitalized or non-hospitalized) should be representative of the general population, not a specific group with comorbidities   - Studies in adults >18 years can be included if the median age is ≥65 years or there is an indication that the majority of the adults are >65 years   - Method for diagnosing IPD must be clearly stated for a study to be included | - Immunocompromised individuals - Other infectious disease - Sub-population with comorbidities - Studies with missed population that did not report the Spn distribution separately for IPD or segregate the distribution for otherwise healthy individuals from immunocompromised individuals - Studies on other age groups and/or that did not report serotype distribution for the age group of interest - Studies that did not state or clearly state the diagnostic method for IPD |
| **Outcome** | - Spn serotypes post-PCV implementation | - Spn serotypes pre-PCV implementation - Other pathogens - Antibiotic resistant Spn strains |
| **Study design** | - SLRs - Meta-analyses - Observational studies with**:** - At least 30 serotyped isolates - Serotyping on samples obtained from sterile sites, e.g., blood, cerebrospinal fluid, pleural effusion, joint fluid, pericardial fluid, etc. | - Other study designs, including but not limited to RCTs, narrative reviews, opinions, editorials, case reports, carriage studies, genomic studies, animal studies, assay development studies - Studies reporting on <30 serotyped isolates or that do not report the total number of serotyped isolates - Studies that did not report different serotypes or groups for IPD - Quarterly or province-level surveillance report, if annual and/or national report were available - Surveillance reports published in surveillance websites - Studies with lack of detailed description about the serotypes of the isolates - Serotyping on samples obtained from non-sterile sites, e.g, nasopharynx or oropharynx, sputum, urine, etc. |
| **Time frame** | 1 January 2006 to 31 December 2020 (SLRs and meta-analyses), or  1 January 2018 to 31 December 2020 (observational studies) | Pre 2006 or post 2020 |
| **Language** | English language | Non-English language |
| **Region** | Global | NA |

# IPD was defined as the isolation of Spn from sterile sites such as blood, cerebrospinal fluids, pleura, effusion, joint fluid or pericardial fluid (1).

IPD, invasive pneumococcal disease; NA, not applicable; PCR, polymerase chain reaction; PCV, pneumococcal conjugate vaccine; RCT, randomized controlled trials; SLR, systematic literature review; Spn, *Streptococcus pneumoniae*

**Table S2.** CAP eligibility criteria

|  | **Inclusion** | **Exclusion** |
| --- | --- | --- |
| **Population** | - CAP^#^ in adults ≥65 years   - The study population (hospitalized or non-hospitalized) should be representative of the general population, not a specific group with comorbidities   - Studies in adults >18 years can be included if the median age is ≥65 years or there is an indication that the majority of the adults are >65 years   - Method for diagnosing CAP must be clearly stated for a study to be included | - Immunocompromised individuals - Other infectious disease - Sub-population with comorbidities - Studies with missed population that did not report the Spn distribution separately for CAP or segregate the distribution for otherwise healthy individuals from immunocompromised individuals - Studies on other age groups and/or that did not report serotypes distribution for the age group of interest - Studies that did not state or clearly state the diagnostic method for CAP |
| **Outcome** | - Spn serotypes post-PCV implementation | - Spn serotypes pre-PCV implementation - Other pathogens - Antibiotic resistant Spn strains |
| **Study design** | - Observational studies with:   - At least 20 serotyped isolates   - Serotyping on samples obtained from non-sterile sites, e.g., sputum, urine, bronchial aspirates, biopsy samples, etc. (non-invasive CAP) | - Other study designs, including but not limited to RCTs, SLR, meta-analysis, narrative review, opinions, editorials, case reports, carriage studies, genomic studies, animal studies, assay development studies - Studies reporting on <20 serotyped isolates or that do not report the total number of serotyped isolates - Studies that did not report different serotypes or groups for CAP - Quarterly or province-level surveillance report, if annual and/or national report were available - Surveillance reports published in surveillance websites - Studies with lack of detailed description about the serotypes of the isolates - Serotyping on samples obtained from sterile sites (invasive CAP) |
| **Time frame** | - 1 January 2006 to 31 December 2020 | - Pre 2006 or post 2020 |
| **Language** | - English language | - Non-English language |
| **Region** | - Global | - NA |

# CAP was defined as pneumonia acquired outside of the hospital (2).

CAP, community-acquired pneumonia; NA, not applicable; PCR, polymerase chain reaction; RCT, randomized controlled trial; SLR, systematic literature review; Spn, *Streptococcus pneumoniae*

## Detailed study selection workflow

Titles and abstracts were screened by two independent reviewers against eligibility criteria. Disagreements were resolved by consensus or reconciliation by a third independent reviewer. Full text screening was performed by a single reviewer. If publications with overlapping data were identified, the publication with the highest number of pneumococcal isolates and/or longest study period was selected.

## Detailed data extraction workflow

Relevant data from individual publications were extracted using DistillerSR (3) by a single reviewer and were confirmed by a second independent reviewer. Extracted data included publication details, study design, data collection period, country or region, clinical manifestation, certainty of diagnosis (i.e., laboratory-confirmed, clinical diagnosis only, mentioning of ‘suspected’ Spn), age of the study population, PCV product used in the country at the time of the study (i.e., PCV7, PHiD-CV, or PCV13), clinical specimen type, serotype detection method, study limitations, study conclusion, total number of isolates serotyped, and number of isolates identified per serotype When the number and/or percentage of serotypes were only reported in a graphical format, the data were extracted using WebPlot Digitizer (4). In addition, information on adult pneumococcal vaccination recommendations and the duration of PCV uptake through infant NIPs during the study period were collected from independent sources.

## Categorization of studies for data analysis

For categorizing studies in vaccine periods (i.e., post-PCV7 and post-PHiD-CV/PCV13). Information provided in the publications were used. When there was no specification of which PCV was used, the PCV period that covered most of the study period was selected. PCV periods that were defined as “transitional” in a study (i.e., a period in which a country was substituting PCV7 for PHiD-CV or PCV13 in the infant NIP), were not included in the analysis.

The primary analyses for both IPD and CAP consisted of studies that were performed in infant NIP context. The selection of studies for these primary analyses was guided by information provided within the publication, as well as the VIEW-hub vaccine information platform from the International Vaccine Access Center (IVAC) (5). Studies that reported a breakdown of serotyping results across different phases of PCV implementation (i.e., licensed use, targeted use [reimbursement for patients at risk only], early routine use, and late routine use) were also considered in these primary analyses; but only the data from early and late PCV periods were considered, as defined in the respective publication.

# Supplementary results

**Table S3**. Serotype distribution in IPD among adults ≥65 years of age post-PHiD-CV/PCV13 implementation through infant national immunization programs (n=12)

| Serotype | Samples identified^#^ | Total samples serotyped^*^ | Pooled percentage average^†^ | Number of studies |
| --- | --- | --- | --- | --- |
| 3 | 2,967 | 25,469 | 11.6 | 12 |
| 8 | 2,456 | 24,479 | 10.0 | 9 |
| 22F | 1,337 | 16,371 | 8.2 | 11 |
| 19A | 1,806 | 25,469 | 7.1 | 12 |
| 12F | 1,473 | 24,861 | 5.9 | 11 |
| 9N | 1,166 | 22,690 | 5.1 | 8 |
| 15A | 726 | 14,649 | 5.0 | 9 |
| 7F | 614 | 13,857 | 4.4 | 7 |
| 6C | 505 | 11,702 | 4.3 | 8 |
| 23A | 553 | 14,107 | 3.9 | 8 |
| 11A | 521 | 14,649 | 3.6 | 9 |
| 33F | 375 | 13,520 | 2.8 | 7 |
| 16F | 341 | 13,005 | 2.6 | 6 |
| 35B | 323 | 12,868 | 2.5 | 7 |
| 23B | 287 | 12,463 | 2.3 | 5 |
| 10A | 321 | 14,649 | 2.2 | 9 |
| 14 | 231 | 12,229 | 1.9 | 8 |
| 15B | 60 | 3,292 | 1.8 | 5 |
| 19F | 193 | 11,702 | 1.6 | 8 |
| 20 | 155 | 9,921 | 1.6 | 6 |
| 6A | 139 | 9,876 | 1.4 | 6 |
| 34 | 125 | 9,334 | 1.3 | 5 |
| 24F | 180 | 14,649 | 1.2 | 9 |
| 38 | 114 | 9,334 | 1.2 | 5 |
| 6B | 98 | 9,699 | 1.0 | 5 |
| 9V | 70 | 9,699 | 0.7 | 5 |
| 23F | 70 | 9,876 | 0.7 | 6 |

# Total number of samples that were identified with the corresponding serotype across all included studies. References: (6-17)

* Total number of samples that were serotyped across all studies that reported the corresponding serotype.

† A pooled percentage average was calculated for each serotype by dividing ‘Samples identified’ by ‘Total samples serotyped’, multiplied by 100.

IPD, invasive pneumococcal disease; n, number of studies included in analysis; PCV13, 13-valent pneumococcal conjugate vaccine; PHiD-CV, pneumococcal non-typeable *Haemophilus influenzae* protein D conjugate vaccine.

**Table S4.** Serotype distribution in CAP among adults ≥65 years of age post- PCV13 implementation through infant national immunization programs (n=8)

| Serotype | Samples identified^#^ | Total samples serotyped^*^ | Pooled percentage average^†^ | Number of studies |
| --- | --- | --- | --- | --- |
| 3 | 446 | 2,691 | 16.6 | 8 |
| 8 | 210 | 2,371 | 8.9 | 6 |
| 19A | 226 | 2,691 | 8.4 | 8 |
| 7F | 130 | 2,632 | 4.9 | 7 |
| 15A | 110 | 2,312 | 4.8 | 5 |
| 5 | 100 | 2,103 | 4.8 | 5 |
| 11A | 96 | 2,312 | 4.2 | 5 |
| 6A | 67 | 1,708 | 3.9 | 7 |
| 12F | 73 | 2,114 | 3.5 | 5 |
| 22F | 79 | 2,371 | 3.3 | 6 |
| 1 | 77 | 2,375 | 3.2 | 6 |
| 23F | 79 | 2,691 | 2.9 | 8 |
| 14 | 61 | 2,434 | 2.5 | 7 |
| 9N | 54 | 2,312 | 2.3 | 5 |
| 19F | 58 | 2,691 | 2.2 | 8 |
| 33F | 46 | 2,371 | 1.9 | 6 |
| 35B | 43 | 2,312 | 1.9 | 5 |
| 4 | 40 | 2,434 | 1.6 | 7 |
| 9V | 42 | 2,691 | 1.6 | 8 |
| 18C | 36 | 2,375 | 1.5 | 6 |
| 17F | 32 | 2,114 | 1.5 | 5 |
| 10A | 29 | 2,312 | 1.3 | 5 |
| 6B | 31 | 2,632 | 1.2 | 7 |
| 23A | 24 | 2,312 | 1.0 | 5 |
| 20 | 19 | 2,371 | 0.8 | 6 |

# Total number of samples that were identified with the corresponding serotype across all included studies. References: (18-25)

* Total number of samples that were serotyped across all studies that reported the corresponding serotype.

† A pooled percentage average was calculated for each serotype by dividing ‘Samples identified’ by ‘Total samples serotyped’, multiplied by 100.

CAP, community-acquired pneumonia; n, number of studies included in analysis; PCV13, 13-valent pneumococcal conjugate vaccine.

Figure S1. Serotype distribution in IPD among adults ≥65 years of age post-PHiD-CV/PCV13 uptake in infants (either through infant national immunization programs or private markets) (n=14)


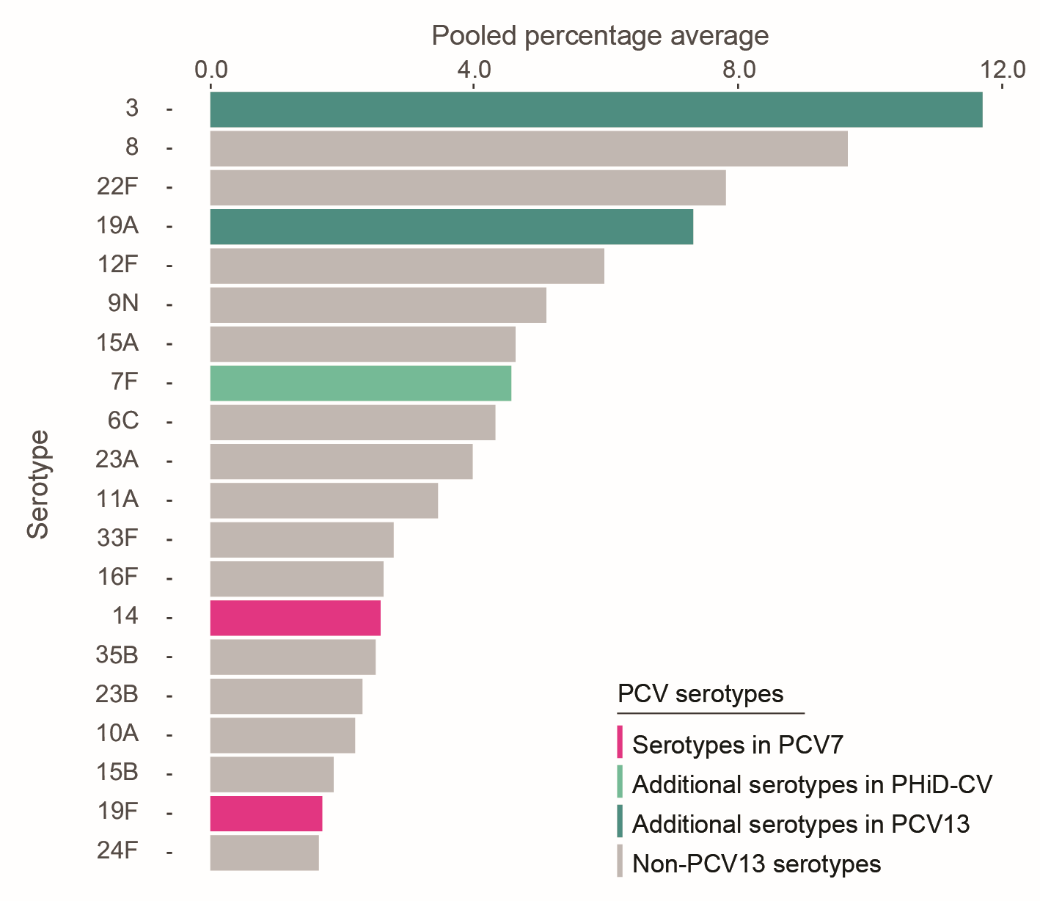


Note: The top 20 serotypes are shown. Serotypes are represented by colors corresponding to the lowest valency PCV product in which they are included. In the PCV legend, the additional serotypes included in the product are relative to the next lower valency product. Pooled percentage averages were calculated for each serotype individually, thus the sum of all serotypes may exceed 100%. Serotype-specific pooled percentage averages were calculated only if 5 or more studies reported on the respective serotype.

IPD, invasive pneumococcal disease; n, number of studies included in analysis; PCV, pneumococcal conjugate vaccine; PCV7, 7-valent PCV; PCV13, 13-valent PCV; PHiD-CV, pneumococcal non-typeable *Haemophilus influenzae* protein D conjugate vaccine.

**Figure S2.** Serotype distribution in IPD among adults ≥65 years of age post-PHiD-CV implementation through the infant national immunization program (n=1 study)


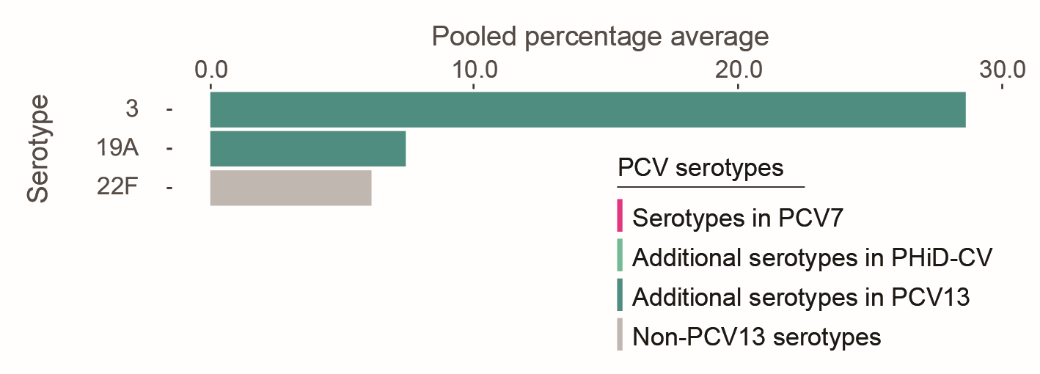


Note: Serotypes are represented by colors corresponding to the lowest valency PCV product in which they are included. In the PCV legend, the additional serotypes included in the product are relative to the next lower valency product. Pooled percentage averages were calculated for each serotype individually, thus the sum of all serotypes may exceed 100%.

IPD, invasive pneumococcal disease; n, number of studies included in analysis; PCV, pneumococcal conjugate vaccine; PCV7, 7-valent PCV; PCV13, 13-valent PCV; PHiD-CV, pneumococcal non-typeable *Haemophilus influenzae* protein D conjugate vaccine.

**Figure S3.** Serotype distribution in IPD among adults ≥65 years of age post-PCV7 implementation through infant national immunization programs (n=3 studies)


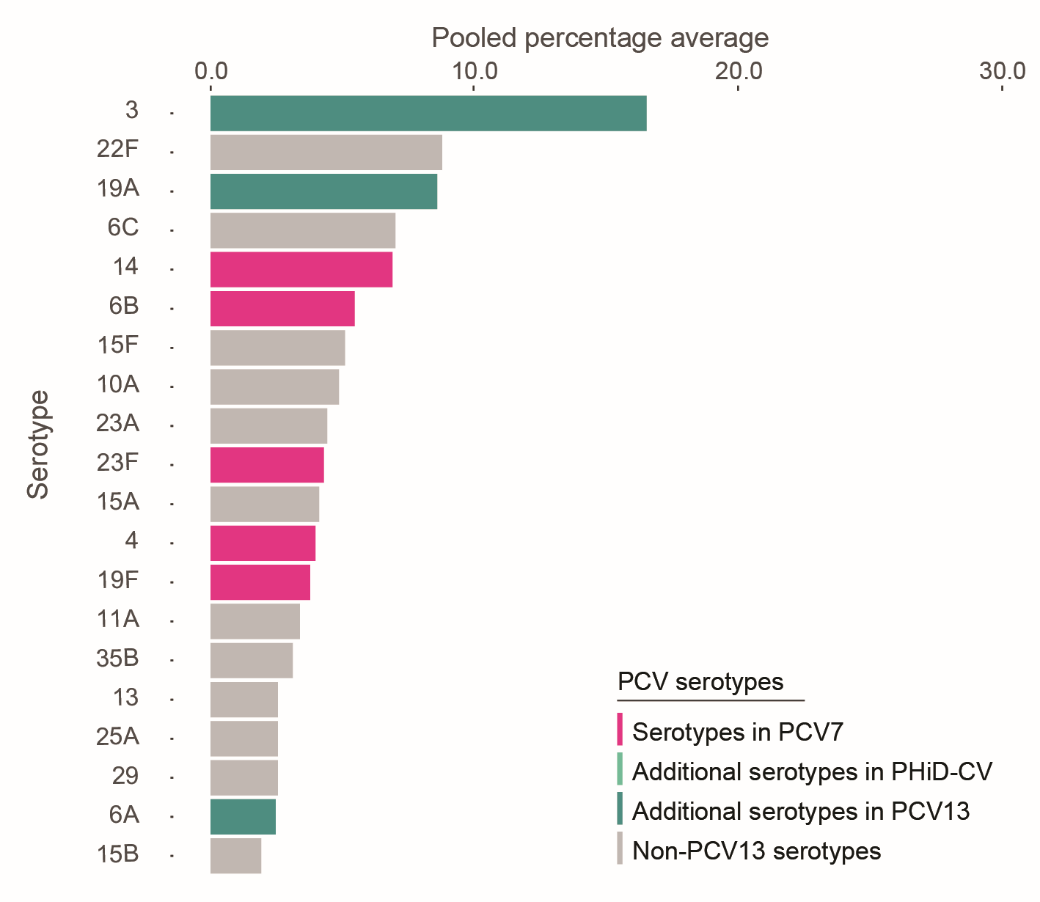


Note: The top 20 serotypes are shown. Serotypes are represented by colors corresponding to the lowest valency PCV product in which they are included. In the PCV legend, the additional serotypes included in the product are relative to the next lower valency product. Pooled percentage averages were calculated for each serotype individually, thus the sum of all serotypes may exceed 100%. Serotype-specific pooled percentage averages were calculated only if 5 or more studies reported on the respective serotype.

IPD, invasive pneumococcal disease; n, number of studies included in analysis; PCV, pneumococcal conjugate vaccine; PCV7, 7-valent PCV; PCV13, 13-valent PCV; PHiD-CV, pneumococcal non-typeable *Haemophilus influenzae* protein D conjugate vaccine.

**Figure S4.** Serotype distribution in CAP among adults ≥65 years of age post-PCV13 uptake in infants (either through infant national immunization programs or private markets) (n=11 studies)


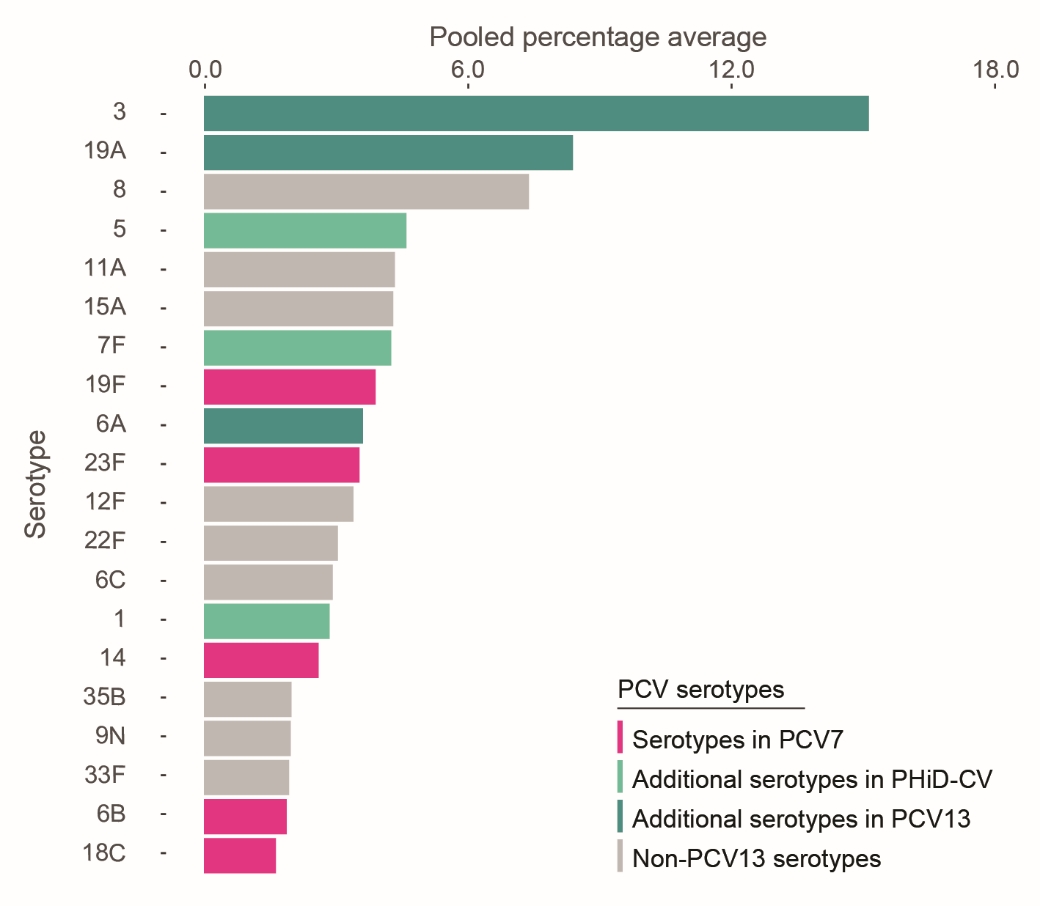


Note: The top 20 serotypes are shown. Serotypes are represented by colors corresponding to the lowest valency PCV product in which they are included. In the PCV legend, the additional serotypes included in the product are relative to the next lower valency product. Pooled percentage averages were calculated for each serotype individually, thus the sum of all serotypes may exceed 100%. Serotype-specific pooled percentage averages were calculated only if 5 or more studies reported on the respective serotype.

CAP, community-acquired pneumonia; n, number of studies included in analysis; PCV, pneumococcal conjugate vaccine; PCV7, 7-valent PCV; PCV13, 13-valent PCV; PHiD-CV, pneumococcal non-typeable *Haemophilus influenzae* protein D conjugate vaccine.

**Figure S5.** Serotype distribution in CAP among adults ≥65 years of age post-PCV7 implementation through infant national immunization programs (n=3 studies)


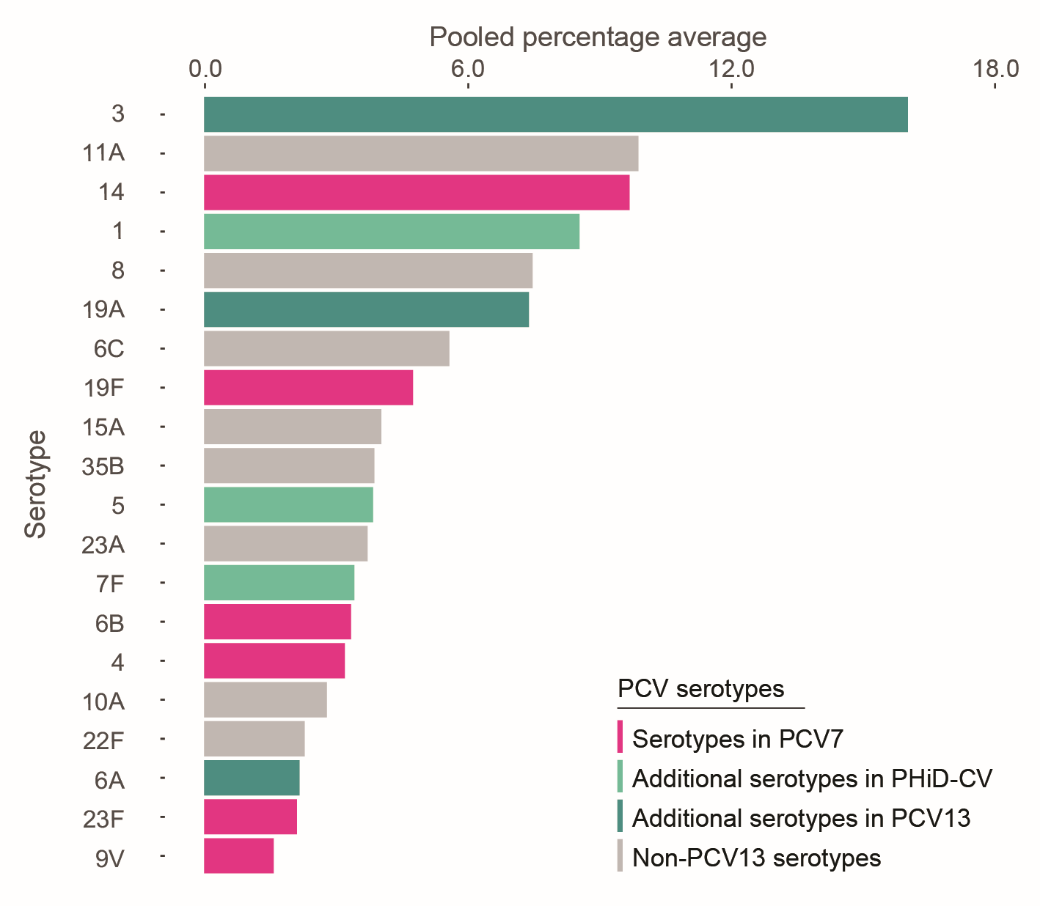


Note: The top 20 serotypes are shown. Serotypes are represented by colors corresponding to the lowest valency PCV product in which they are included. In the PCV legend, the additional serotypes included in the product are relative to the next lower valency product. Pooled percentage averages were calculated for each serotype individually, thus the sum of all serotypes may exceed 100%.

CAP, community-acquired pneumonia; n, number of studies included in analysis; PCV, pneumococcal conjugate vaccine; PCV7, 7-valent PCV; PCV13, 13-valent PCV; PHiD-CV, pneumococcal non-typeable *Haemophilus influenzae* protein D conjugate vaccine.

# References

1. Randle E, Ninis N, Inwald D. Invasive pneumococcal disease. Arch Dis Child Educ Pract Ed (2011) 96:183-90. doi: 10.1136/adc.2010.191718

2. NICE. Clinical guideline: Pneumonia scope. (2012). <https://www.nice.org.uk/guidance/cg191/documents/pneumonia-final-scope2>. [Accessed April 14, 2025].

3. DistillerSR. <https://www.distillersr.com/>. [Accessed 23/05/2024].

4. WebPlot Digitizer. <https://automeris.io/WebPlotDigitizer>. [Accessed April 14, 2025].

5. International Vaccine Access Center (IVAC), Johns Hopkins Bloomberg School of Public Health. VIEW-hub. <https://view-hub.org/vaccine/pcv>. [Accessed 23/05/2024].

6. Amin-Chowdhury Z, Collins S, Sheppard C, Litt D, Fry NK, Andrews N, et al. Characteristics of invasive pneumococcal disease caused by emerging serotypes after the introduction of the 13-valent pneumococcal conjugate vaccine in England: A prospective observational cohort study, 2014-2018. Clin Infect Dis (2020) 71:e235-e43. doi: 10.1093/cid/ciaa043

7. Ciruela P, Broner S, Izquierdo C, Pallares R, Munoz-Almagro C, Hernandez S, et al. Indirect effects of paediatric conjugate vaccines on invasive pneumococcal disease in older adults. Int J Infect Dis (2019) 86:122-30. doi: 10.1016/j.ijid.2019.06.030

8. Danis K, Varon E, Lepoutre A, Janssen C, Forestier E, Epaulard O, et al. Factors associated with severe nonmeningitis invasive pneumococcal disease in adults in France. Open Forum Infect Dis (2019) 6:ofz510. doi: 10.1093/ofid/ofz510

9. de Miguel S, Domenech M, Gonzalez-Camacho F, Sempere J, Vicioso D, Sanz JC, et al. Nationwide trends of invasive pneumococcal disease in Spain from 2009 through 2019 in children and adults during the pneumococcal conjugate vaccine era. Clin Infect Dis (2021) 73:e3778-e87. doi: 10.1093/cid/ciaa1483

10. Demczuk WHB, Martin I, Desai S, Griffith A, Caron-Poulin L, Lefebvre B, et al. Serotype distribution of invasive Streptococcus pneumoniae in adults 65 years of age and over after the introduction of childhood 13-valent pneumococcal conjugate vaccination programs in Canada, 2010-2016. Vaccine (2018) 36:4701-7. doi: 10.1016/j.vaccine.2018.06.018

11. Fenoll A, Ardanuy C, Linares J, Cercenado E, Marco F, Fleites A, et al. Serotypes and genotypes of S. pneumoniae isolates from adult invasive disease in Spain: A 5-year prospective surveillance after pediatric PCV13 licensure. The ODIN study. Vaccine (2018) 36:7993-8000. doi: 10.1016/j.vaccine.2018.10.098

12. Ladhani SN, Collins S, Djennad A, Sheppard CL, Borrow R, Fry NK, et al. Rapid increase in non-vaccine serotypes causing invasive pneumococcal disease in England and Wales, 2000-17: a prospective national observational cohort study. Lancet Infect Dis (2018) 18:441-51. doi: 10.1016/S1473-3099(18)30052-5

13. Park DC, Kim SH, Yong D, Suh IB, Kim YR, Yi J, et al. Serotype distribution and antimicrobial resistance of invasive and noninvasive Streptococcus pneumoniae isolates in Korea between 2014 and 2016. Ann Lab Med (2019) 39:537-44. doi: 10.3343/alm.2019.39.6.537

14. Richter L, Schmid D, Kanitz EE, Zwazl I, Pollabauer E, Jasinska J, et al. Invasive pneumococcal diseases in children and adults before and after introduction of the 10-valent pneumococcal conjugate vaccine into the Austrian national immunization program. PLoS One (2019) 14:e0210081. doi: 10.1371/journal.pone.0210081

15. Ubukata K, Takata M, Morozumi M, Chiba N, Wajima T, Hanada S, et al. Effects of pneumococcal conjugate vaccine on genotypic penicillin resistance and serotype changes, Japan, 2010-2017. Emerg Infect Dis (2018) 24:2010-20. doi: 10.3201/eid2411.180326

16. Yanagihara K, Kosai K, Mikamo H, Mukae H, Takesue Y, Abe M, et al. Serotype distribution and antimicrobial susceptibility of Streptococcus pneumoniae associated with invasive pneumococcal disease among adults in Japan. Int J Infect Dis (2021) 102:260-8. doi: 10.1016/j.ijid.2020.10.017

17. Zintgraff J, Fossati S, Pereira CS, Veliz O, Regueira M, Moscoloni MA, et al. Distribution of PCV13 and PPSV23 Streptococcus pneumoniae serotypes in Argentinean adults with invasive disease, 2013-2017. Rev Argent Microbiol (2020) 52:189-94. doi: 10.1016/j.ram.2019.11.004

18. Benfield T, Skovgaard M, Schonheyder HC, Knudsen JD, Bangsborg J, Ostergaard C, et al. Serotype distribution in non-bacteremic pneumococcal pneumonia: association with disease severity and implications for pneumococcal conjugate vaccines. PLoS One (2013) 8:e72743. doi: 10.1371/journal.pone.0072743

19. Forstner C, Kolditz M, Kesselmeier M, Ewig S, Rohde G, Barten-Neiner G, et al. Pneumococcal conjugate serotype distribution and predominating role of serotype 3 in German adults with community-acquired pneumonia. Vaccine (2020) 38:1129-36. doi: 10.1016/j.vaccine.2019.11.026

20. Huijts SM, Pride MW, Vos JM, Jansen KU, Webber C, Gruber W, et al. Diagnostic accuracy of a serotype-specific antigen test in community-acquired pneumonia. Eur Respir J (2013) 42:1283-90. doi: 10.1183/09031936.00137412

21. Isturiz RE, Ramirez J, Self WH, Grijalva CG, Counselman FL, Volturo G, et al. Pneumococcal epidemiology among us adults hospitalized for community-acquired pneumonia. Vaccine (2019) 37:3352-61. doi: 10.1016/j.vaccine.2019.04.087

22. LeBlanc J, ElSherif M, Ye L, MacKinnon-Cameron D, Ambrose A, Hatchette TF, et al. Age-stratified burden of pneumococcal community acquired pneumonia in hospitalised Canadian adults from 2010 to 2015. BMJ Open Respir Res (2020) 7. doi: 10.1136/bmjresp-2019-000550

23. Pick H, Daniel P, Rodrigo C, Bewick T, Ashton D, Lawrence H, et al. Pneumococcal serotype trends, surveillance and risk factors in UK adult pneumonia, 2013-18. Thorax (2020) 75:38-49. doi: 10.1136/thoraxjnl-2019-213725

24. Prato R, Fortunato F, Cappelli MG, Chironna M, Martinelli D. Effectiveness of the 13-valent pneumococcal conjugate vaccine against adult pneumonia in Italy: a case-control study in a 2-year prospective cohort. BMJ Open (2018) 8:e019034. doi: 10.1136/bmjopen-2017-019034

25. Sando E, Suzuki M, Furumoto A, Asoh N, Yaegashi M, Aoshima M, et al. Impact of the pediatric 13-valent pneumococcal conjugate vaccine on serotype distribution and clinical characteristics of pneumococcal pneumonia in adults: The Japan Pneumococcal Vaccine Effectiveness Study (J-PAVE). Vaccine (2019) 37:2687-93. doi: 10.1016/j.vaccine.2019.04.009
